# Insights into regulatory T-cell and type-I interferon roles in determining abacavir-induced hypersensitivity or immune tolerance

**DOI:** 10.3389/fimmu.2025.1612451

**Published:** 2025-06-06

**Authors:** Marco Cardone, Hratch M. Baghdassarian, Maryam Khalaj, Kirthiram Krishnaveni Sivakumar, SuJin Hwang, Sintayehu Gebreyohannes, Kazuyo Takeda, Yura Jang, Nathan E. Lewis, Michael A. Norcross, Montserrat Puig

**Affiliations:** ^1^ Division of Pharmaceutical Quality Research IV, Office of Pharmaceutical Quality Research, Office of Pharmaceutical Quality, Center for Drug Evaluation and Research, Food and Drug Administration, Silver Spring, MD, United States; ^2^ Departments of Pediatrics and Bioengineering, University of California, San Diego, CA, United States; ^3^ Microscopy and Imaging Core Facility, Center for Biologics Evaluation and Research, Food and Drug Administration, Silver Spring, MD, United States; ^4^ Department of Bioengineering, Technical University of Denmark, Kongens Lyngby, Denmark

**Keywords:** drug hypersensitivity reactions, abacavir, Treg, type-I IFN, T-cell, HLA, immune tolerance, innate immunity

## Abstract

**Introduction:**

Clinical use of several small molecule drugs may lead to severe T-cell-mediated idiosyncratic drug hypersensitivity reactions (iDHR) linked to HLA alleles, including abacavir (ABC) with HLA-B*57:01. Due to study limitations in humans, pathogenic networks in iDHR remain elusive. HLA transgenic murine models have been proposed to bridge knowledge gaps in tolerance and susceptibility to drugs.

**Methods:**

Mice expressing HLA-B*57:01 and Foxp3-DTR/EGFP were generated to selectively deplete regulatory T-cells (Treg) with diphtheria toxin. ABC was administered for 8 days alone or together with cell- and cytokine-depleting antibodies. Cellular and transcriptomic responses were analyzed by RNA, flow cytometry and fluorescence methods.

**Results:**

While CD8^+^ T-cell responses to ABC require HLA presentation, ABC also triggered mitochondrial stress in macrophages *in vitro*, independently of HLA. *In vivo*, Treg were the primary mechanism of drug tolerance controlling HLA presentation and costimulation by antigen presenting cells. Treg ablation uncovered immune adverse events linked to activation and proliferation of both drug-specific and bystander CD8^+^ T-cells through CD28-mediated pathways with support from CD4^+^ non-Treg. Type-I interferon (IFN-I) and cellular-stress pathways influenced the fate of lymph node cells responding to ABC, implicating innate immune cells such as macrophages and plasmacytoid dendritic cells in the development of T-cell responses against the drug. IFN-I and IL-2 were necessary for CD8^+^ T-cell differentiation and ABC-induced adverse reactions.

**Conclusions:**

This study unveils novel immune mechanisms driven by drug and host-related factors required for *in vivo* reactions and sheds light on potential biomarker and therapeutic targets for managing and preventing severe and life-threatening iDHR.

## Graphical Abstract

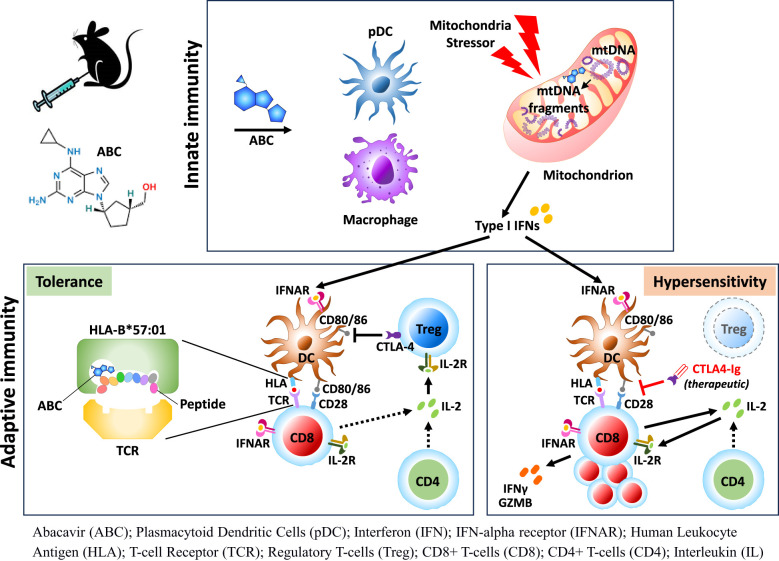

## Introduction

Idiosyncratic and delayed-onset DHR (iDHR) have been linked to the expression of specific HLA alleles and the activation of T-cells that recognize self-epitopes altered by the drug (1, 2). Although generally rare, such autoimmune-like activation can lead to severe and life-threatening clinical outcomes. The reverse transcriptase inhibitor ABC has been selected to study iDHR for its high positive predictive value with the HLA-B*57:01 allele, although the association of HLA expression and disease is not absolute (3, 4). Immune inhibitory mechanisms are thought to counteract effector immune response against the drug (5), however, details of the regulatory networks in iDHR remain elusive due to study limitations in humans.

HLA Tg mice strains have emerged as valuable research options to address critical technical and knowledge gaps. In particular, HLA-B*57:01 Tg mice, despite being tolerant to ABC, can replicate the observed drug hypersensitivity syndrome in humans upon CD4^+^ T-cell ablation (6). Although those studies suggested CD4^+^ T-cells with regulatory function may limit the activation of ABC-reactive CD8^+^ T-cells, the results did not conclusively demonstrate this (6).

While host factors such as HLA risk alleles, TCR clonality, and disease-related aspects can partially explain the idiosyncratic nature of drug-induced adaptive immune responses, the exact trigger is less understood. Drug-associated toxicities can lead to off-target inflammation and stress, including not uncommon cellular damage effects involving mitochondrial membrane leakage, inflammasome activation and innate immune inflammation (7, 8). The emerging role of the innate immune system in iDHR has been previously reviewed (9). However, evidence of iDHR-associated drugs causing innate immune activation is limited and unclear, and very few examples have been reported in the literature (10).

In the present report, we generated a conditional Foxp3 knockout expressing HLA-B*57:01 Tg mice to dissect unreported immune mechanisms driving ABC hypersensitivity. Before administering ABC *in vivo*, we sought evidence of potential cytotoxic effects of the drug on immune cells. Despite of *in vitro* data supporting ABC-induced distress on macrophages, immune competent animals were tolerant to drug treatment. In contrast, Foxp3^+^CD4^+^ Treg depletion resulted in ABC-driven CD8^+^ T-cell activation, by eliminating inhibitory binding of CTLA-4 to CD80 and CD86, and by IL-2 sequestration by Treg (11). Transcriptomic studies in LN cells identified key networks implicated in determining the fate of drug-responding CD8^+^ T-cells early in treatment, including cellular stress pathways. A clear IFN-I signature revealed in innate immune cells and T-cells was later found to be critical in supporting the CD8^+^ T-cell response to drug. This study details a novel multifactorial contribution of ABC to iDHR including a potential role in triggering both HLA-independent and dependent immune mechanisms that are tightly regulated by Treg. These pathways could be considered for future therapeutic strategies.

## Materials and methods

### RAW-Blue^TM^ cells

RAW-Blue^TM^ cells (InvivoGen) were cultured with the drug and toll-like receptor agonists prior to assessing NF-kB activation, mRNA expression and mROS and cleaved Caspase-1 detection as indicated in **Supplementary Methods**.

### Mice

DEREG HLA-B*57:01 transgenic mice (hereafter referred as “mice”) were generated by crossing hemizygous C57BL/6-Tg (Foxp3-DTR/EGFP)23.2Spar/Mmjax females with HLA-B*57:01 Tg males (B9 Tg mice) (6) (**Supplementary Figures 1A–D**). Animals were treated with DT (i.p.), ABC (i.p. or topical), and cytokine-depleting antibodies or CTLA-4-Ig, as indicated in **Supplementary Methods**.

### Cell culture

For cell cultures with purified Treg, MACS-purified CD8^+^ T-cells (1.2 x 10^6^/mL) were co-cultured with MACS-purified CD4^+^CD25^+^ natural Treg (Miltenyi Biotec) at different ratios in RPMI-10 media [RPMI 1640 supplemented with 2 mM L-glutamine, 1 mM sodium pyruvate, 1X MEM non-essential amino acids, 1X MEM vitamin solution, 10 mM Hepes, 0.05 mM 2β-ME, 100 U/mL penicillin, and 100 μg/mL Streptomycin (all from GIBCO)], in the presence or absence of ABC. For intracellular detection of IFN-γ or granzyme B (GZMB), 2 x 10^6^ splenocyte cultures were prepared and treated as previously described (6).

### Gene expression

LNs or spleen sections were submerged in TRIzol (Invitrogen), flash-frozen, and stored at -80°C until processing for RNA extraction as previously described (6). Total RNA from tissue homogenates or from RAW-Blue^TM^ cells was extracted following the TRIzol protocol and reversed transcribed using the High-Capacity cDNA RT Kit (Applied Biosystems) as per manufacturer’s instructions. Gene amplification was performed using TLDA mouse immune array or specific gene expression assays on a Viia7 real-time PCR system (all from Applied Biosystems) as in (6). ΔΔCt method was used to calculate the fold changes normalizing against GAPDH.

### Flow cytometry

Flow cytometry was used to phenotype mice, and immune cell subset analysis by intracellular and/or surface marker staining as further detailed in the **Supplementary Methods**.

### Single-cell RNA sequencing

Prior to testing for scRNAseq, samples were depleted of B-cells (130-121-301, Miltenyi Biotec), and enriched with Pan DCs (130-100-875, Miltenyi Biotec) to enhance detection of DC transcriptomes (except for untreated sample due to low levels of Pan DC recovered). Ten-thousand LN cells per sample were processed using a 10X Genomics controller and the Chromium next GEM single cell 3’ kit (v.3.1) (#1000130, 10X Genomics). Resulting libraries were sequenced with the Illumina NovaSeq SP kit. Default parameters were used unless otherwise stated. Data processing and analysis are detailed in **Supplementary Methods**. Original code has been deposited at https://doi.org/10.5281/zenodo.15532562.

### Cell depletion

To deplete plasmacytoid DC or macrophage, animals were injected *i.p.* with 0.5 mg of anti-CD317 mAb (αPDCA1, BioXcell) or 100 mg of clodronate sulfate liposomes (LIPOSOMA) 3 days prior to ABC treatment and subsequently at days 1, 3, and 5 (pDC) or days 1, 5, and 7 (Mf), respectively.

## Results

### ABC induces IFN-I and *Il1b* in macrophages through cellular-stress-related pathways in an HLA-independent manner

**Figure 1:**
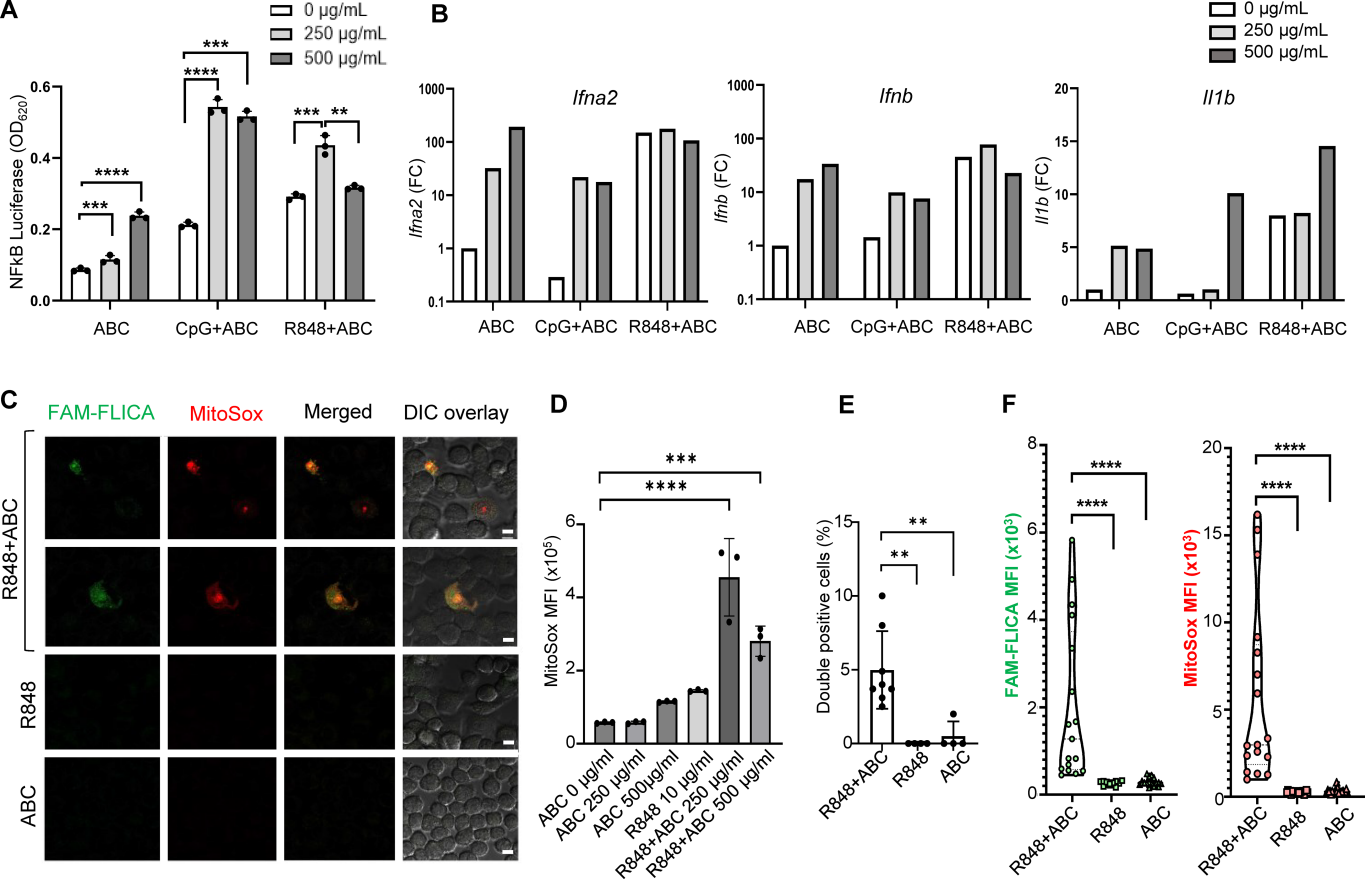
ABC induction of innate immune activation through cellular stress pathways in an HLA-independent manner. Murine macrophage RAW-Blue^TM^ cells, lacking HLA-B*57:01, were cultured with drug and TLR agonists. **(A)** NFκB activation by ABC and/or CpG1555 ODN (100 nM), or R848 (10 µg/mL). **(B)** IFN-I and *Il1b* gene expression by Taqman [pooled replicate wells in **(A)**]. This is a representative independent experiment out of two with comparable results (see **Supplementary Figure 2A**). **(C)** Representative image of immunostaining showing caspase activation (FAM-FLICA) and ROS production (MitoSox) upon treatment with ABC (250 µg/mL) or/and R848 (10 µg/mL) (scale bar - 10 µm). **(D)** ABC dose finding for MitoSox mean fluorescence intensity (MFI). **(E)** Double positive cells. **(F)** MFI of FAM-FLICA and MitoSox in 5 microscopy fields from **(C)**. Data points represent individual readouts, bars show mean ± SD. Statistics: **(D)** one-way ANOVA; ***P < 0.0005, and ****P < 0.0001; **(A, E, F)** unpaired, 2-tailed Student’s t-test; **P < 0.005, **P < 0.005, and ****P < 0.0001.

First, to explore the cytotoxic potential of ABC, murine macrophages (RAW-Blue^TM^ cell line) were exposed to different concentrations of drug *in vitro*, in the presence or absence of innate immune activators. Macrophages responded to ABC in a dose-dependent manner (**Figure 1A**). Synergistic effects with toll-like receptor (TLR) agonists were observed at drug concentrations between 250-500 µg/mL. Additionally, ABC increased transcription of IFN-I genes and *Il1b* although additive effects with TLR agonists occurred only for *Il1b* levels at a higher drug concentration (**Figure 1B**). Macrophages exposed to ABC showed signs of mitochondrial dysfunction [reactive oxygen species (ROS)] and caspase-related inflammasome activation (**Figures 1C–F**; **Supplementary Figure 2**) when combined with TLR7/8 agonist R848 (**Figure 1D**), as previously shown for human THP-1 macrophages (12). Data from these orthogonal methods support the activation of macrophages by ABC through the activation of innate immune and cell-stress pathways, occurring in an HLA-independent manner. Determining whether primary macrophages would have comparable *in vitro* reactions to ABC will require further studies.

### Treg-depleted mice exhibit severe hypersensitivity symptoms by day 8 of ABC exposure

**Figure 2:**
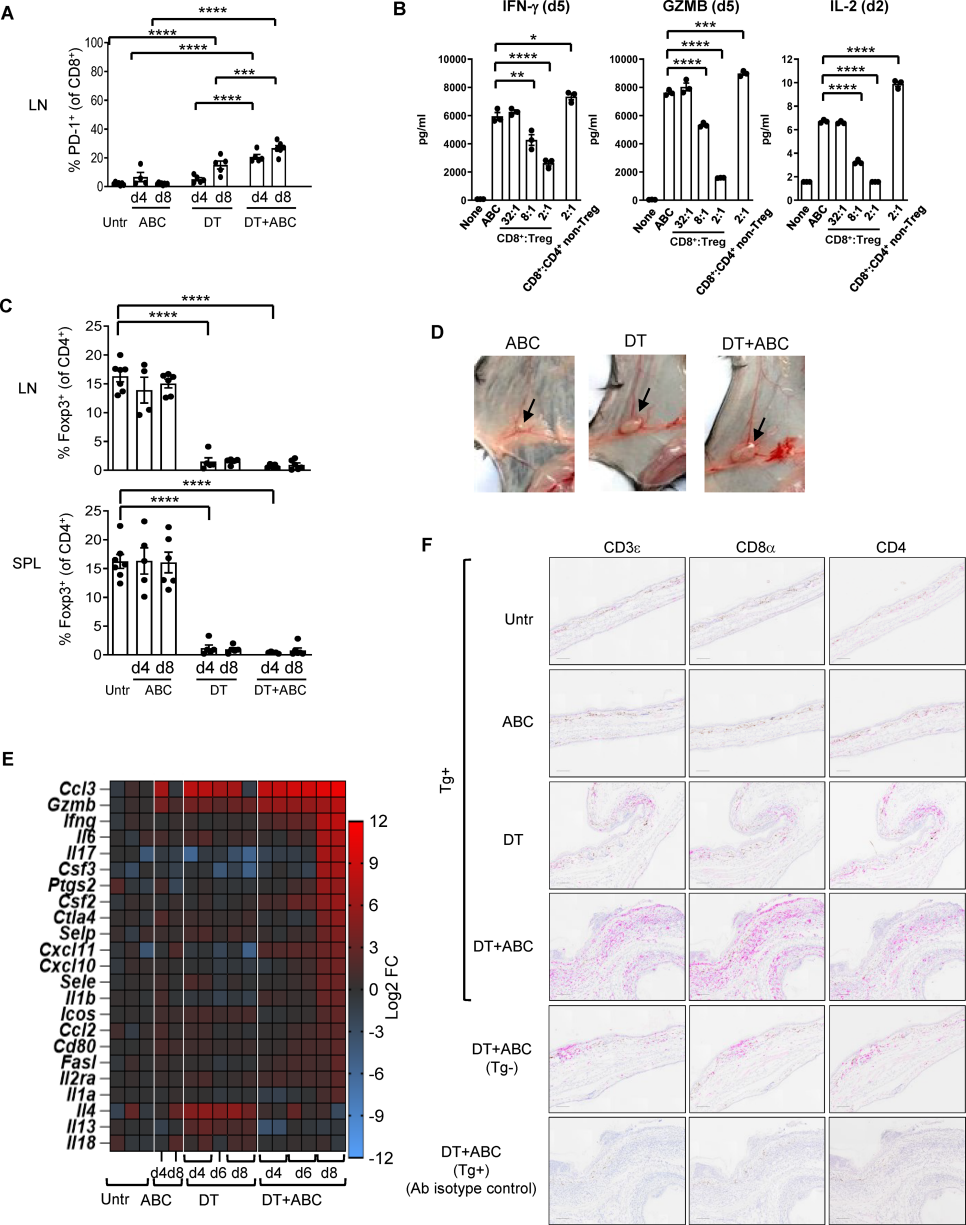
Treg control of ABC-induced effector CD8+ T-cells. Mice were treated with ABC, DT or DT+ABC as detailed in the materials and methods. **(A)** CD8^+^ T-cells activation in lymphoid nodes (LN) at day (d) 4 and 8 of treatment (n=7–14 mice/group). **(B)** Drug-naïve splenic CD8^+^ T-cells were cultured with ABC and at different ratios with CD25^+^ Treg or CD4^+^ non-Treg. **(C)** Depletion of Treg (Foxp3^+^CD4^+^ T-cells) in spleen (SPL) and LN after DT and/or ABC administration versus untreated (Untr). Black dots represent mice from multiple experiments. Data are represented as mean ± SEM. **(D)** Lymphadenopathy at d8 of treatment. **(E)** Fold-increase expression of immune-related markers in LN cells of treated versus untreated animals overtime by real-time PCR. **(F)** Representative IHC staining of CD3ε, CD8α and CD4 cells in skin tissue sections of mice from different treatment groups. Tg- mice treated with DT+ABC were used as controls for HLA specificity. Negative control corresponds to sections stained with isotype control antibodies (Ab). Scale bar = 100µm. Statistics **(A–C)**: one-way ANOVA; *P < 0.05, **P < 0.005, ***P < 0.0005 and ****P < 0.0001.

Eight-day administration of ABC to mice led to drug tolerance (**Figure 2A**), indicating that the observed cytotoxic effects of ABC *in vitro* were insufficient to trigger adaptive immune responses *in vivo* and implicating a role for Treg cells in drug tolerance. To confirm this hypothesis, *in vitro* co-culture experiments showed drug-naïve natural Treg, unlike non-Treg CD4^+^ T-cells, reduced cytokine production of CD8^+^ T-cells responding to ABC (**Figure 2B**). Based on this result, the role of Treg in controlling ABC reactions *in vivo* was subsequently investigated. Treg were rapidly depleted from lymphoid organs and blood upon diphtheria toxin (DT) administration (**Figure 2C**), resulting in generalized drug-independent lymphadenopathy by day (d)8 (**Figure 2D**). Co-administration of DT+ABC led to expansion of CD8^+^ PD1^+^ T-cells in the LN (**Figure 2A**) as well as elevated expression of inflammatory genes (**Figure 2E**). The animals eventually developed severe cytokine storm-like syndrome (**Supplementary Figure 3**). DT+ABC mice exhibited robust dermal and epidermal CD8^+^ T-cell infiltration (**Figure 2F**) associated with ear thickening. CD4^+^ T-cells were also observed in all Treg-depleted animals (**Figure 2F**).

### Treg limit systemic accumulation of ABC and self-reactive PD-1^+^ T-cells

**Figure 3:**
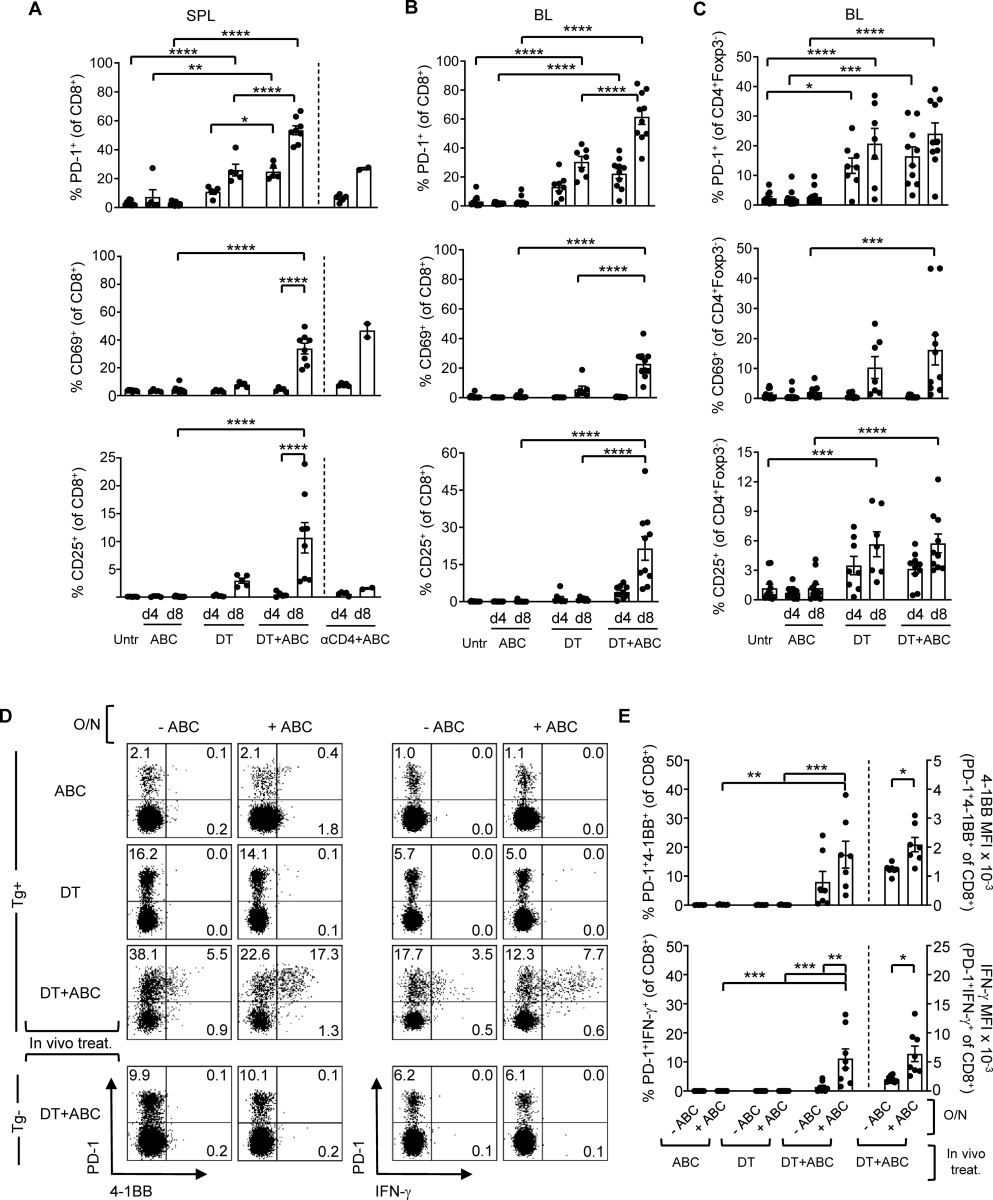
Depletion of Treg leads to expansion of ABC-reactive PD-1+CD8+ T-cells and self-reactive CD4+ non-Treg. Animals were treated with ABC, DT or a combination of DT+ABC as detailed in the materials and methods. In certain experiments, anti-CD4 depleting antibody (αCD4) was used as control. **(A)** Activation markers on CD8^+^ T-cells in spleen (SPL) at d4 and 8 of treatment (n=7–14 mice/group). **(B, C)** Percent of activated CD8^+^ T-cells **(B)** and CD4^+^ non-Treg cells **(C)** in the blood (BL) of animals in **(A)**. **(D)** Representative dot plots with expression levels of effector molecules (4-1BB and IFN-γ) in splenic CD8^+^PD-1^+^ T-cells upon a subsequent overnight (O/N) stimulation with 10 µg/mL of ABC *in vitro*. Tg- animals were used as negative control for the DT+ABC group. **(E)** Percent of activated CD8^+^PD-1^+^ T-cells (left-axis) and expression levels of effector molecules (MFI) (right-axis) for all animals in experiment **(C)**. Summary data are represented as mean ± SEM. Statistics for **(A–C, E)** % 4-1BB^+^ and IFN-γ^+^ cells: one-way ANOVA; *P < 0.05, **P < 0.005, ***P < 0.0005, and ****P < 0.0001. Statistics for **(E)** % 4-1BB^+^ and % IFN-γ^+^ MFI: unpaired, 2-tailed Student’s t-test; *P < 0.05, **P < 0.005, ***P < 0.0005, and ****P < 0.0001.

In addition to the LN, activated CD8^+^ PD1^+^ T-cells also accumulated in spleen (**Figure 3A**) and blood (**Figure 3B**) of DT and DT+ABC mice over time. However, DT+ABC mice exhibited higher frequencies of cells expressing PD-1^+^, CD69^+^ and CD25^+^ (**Figures 3A, B**) possibly due to coexistence of both drug antigen-specific and self-reactive cells. Treg ablation also enhanced the expression of activated self-reactive CD4^+^ non-Treg (Foxp3^-^) independently of ABC (**Figure 3C**). Since the drug-specific CD8^+^ T-cell response occurred faster in DT+ABC than αCD4+ABC mice lacking CD4^+^ T-cells, we hypothesized that self-reactive CD4^+^ T-cells may enhance the ABC response in DT+ABC animals (**Figure 3A**).

At d4 of treatment, ABC, DT and DT+ABC mice showed early activated CD8^+^ T-cells (PD-1^-^CD69^+^CD25^-^) only in the LN, along with a more functionally advanced subset (PD-1^+^CD69^-^CD25^+^) enriched in DT+ABC, also present in spleen (**Supplementary Figure 4**). PD-1^+^CD69^+^ cells could be in a transitioning state. Later, by d8, enriched LN and splenic CD8^+^PD-1^+^ T-cells in DT+ABC mice exhibited effector-like phenotype (CD44^hi^CD62L^-^) and proliferative capacity (Ki-67^+^) (**Supplementary Figures 5A, 6A**) unlike the early-activated CD8^+^PD-1^-^CD69^+^ T-cell population (gray population in **Supplementary Figures 5B, 6B**). The majority of CD25^+^ cells, as well as those expressing terminal differentiation markers were PD-1^+^ (**Supplementary Figures 5C, 6C**). CD8^+^PD-1^+^ T-cells of DT+ABC mice quickly reacted to ABC restimulation in an HLA-B*57:01-dependent manner (**Figures 3D, E**) and were more differentiated (**Supplementary Figure 7**). Importantly, self-reactive CD8^+^PD-1^+^ T-cells, present both in LN and spleen of DT animals and DT+ABC Tg^-^ mice expressed CD44 and Ki-67, but not CD25, suggesting IL-2 independent expansion. These cells expressed less terminally differentiated markers (**Supplementary Figures 5A, 6A**) and failed to respond to ABC re-stimulation *in vitro* (**Figures 3D, E**).

Overall, these data indicate that Treg not only limit CD8^+^ T-cell drug-effector function, but also the activation and expansion of both drug-specific and self-reactive T-cells. Moreover, CD8^+^ T-cell expansion in DT+ABC mice may occur through both IL-2-dependent and independent mechanisms.

### Treg-depleted mice accumulate mature DCs essential for T-cell expansion/effector function

**Figure 4:**
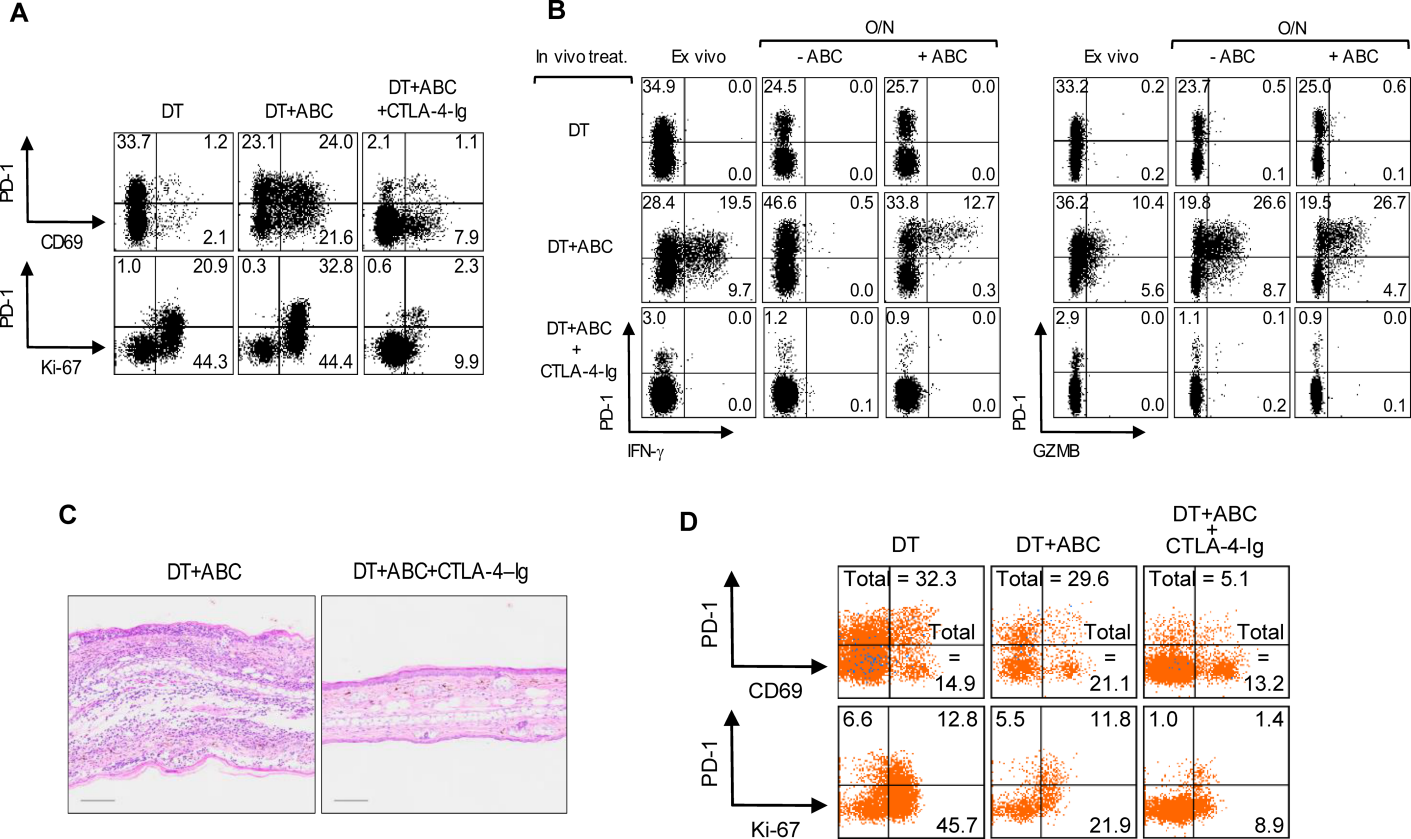
Treg impair optimal costimulation of T-cells by DC during ABC treatment. Mice were treated with DT, or a combination of DT+ABC and/or CTLA-4-Ig as detailed in the materials and methods. LN cells were analyzed by FACS. **(A, B)** Representative dot plots of activation and proliferation markers **(A)** and effector molecules **(B)** in splenic CD8^+^ T-cell *ex vivo* or after overnight (O/N) restimulation with 10 µg/mL of ABC **(B)**. IFN-γ and GZMB were detected by intracellular staining. **(C)** Representative H&E staining of ear tissue section of animals treated with DT+ABC in the absence or presence of CTLA-4–Ig (scale bar = 100µm). **(D)** Representative dot plots of activation markers on splenic CD4^+^ non-T-cell (n=3 mice/group).

Treg depletion is known to increase CD80 and CD86 expression on DCs, which we confirmed in LN and splenic migratory DCs upon DT treatment and was amplified with addition of ABC in both migratory and resident DCs (data not shown). We therefore tested whether blocking DC costimulatory signals with CTLA4-Ig could prevent CD8^+^ T-cell activation to ABC. CTLA4-Ig lowered the frequency of CD69^+^ and proliferating CD8^+^PD-1^+^Ki-67^+^ T-cells in DT+ABC mice to levels comparable to or below those found in DT animals (**Figure 4A**), impaired their effector potential in recall assays, and eliminated skin inflammation (**Figures 4B, C**). Interfering with costimulation affected the expansion of both self-reactive and drug-responding CD8^+^PD-1^+^ T-lymphocytes, and self-reactive CD4^+^ non-Treg cells (**Figure 4D**). In the absence of Treg, CD80/86 on DCs is available to bind CD28 on T-cells supporting drug-specific T-cell receptor activation signals and the early development of ABC-induced CD8^+^ T-cell responses.

### Single-cell transcriptomic analysis reveals ABC-induced IFN-I networks and key pathways determining the fate of drug-reactive cells

**Figure 5:**
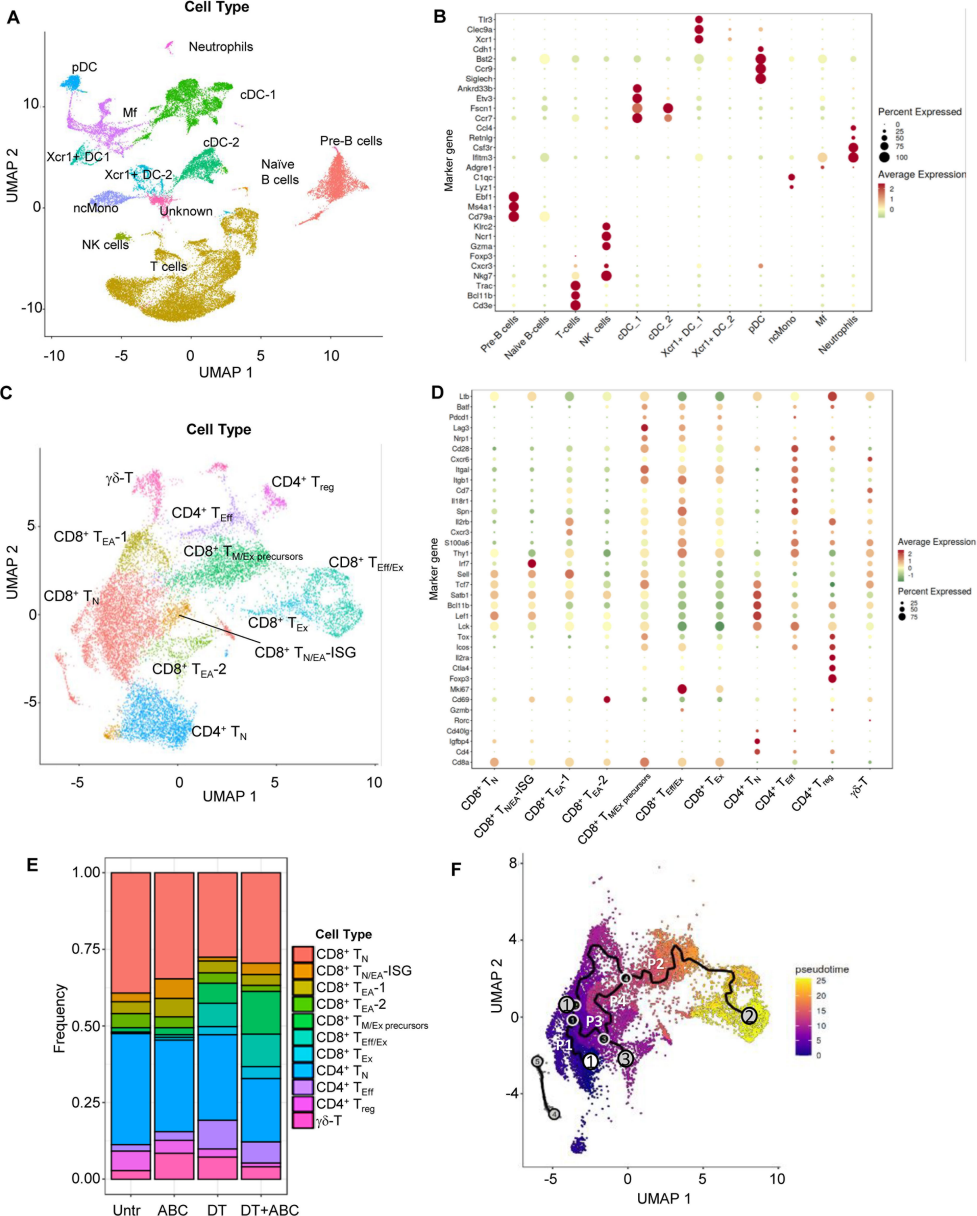
LN single-cell landscape of animals treated with ABC and/or depleted of Treg. LNs were collected at d4 of treatment and processed for scRNA-seq analysis (n=1/group). Data from the 5 samples (Untr, ABC, DT, DT+ABC, αCD4+ABC) were pooled together for integrated, unsupervised analysis. **(A)** Cellular landscape with 13 different cell subtypes including lymphocytes (T, B and NK cells), neutrophils, conventional DC (cDC) and Xcr1-expressing DC (Xcr1+ DC), macrophages (Mf), plasmacytoid DC (pDC) and non-conventional monocytes (ncMono). **(B)** Canonical markers representing each cell type except for the unidentified cluster “Unknown” (see complete gene subset for each cluster in **Supplementary Table 1**). **(C)** Integrated supervised analysis of the T-cells in **(A)** resulted in 11 different CD8^+^, CD4^+^ and γδ-T-cell subsets, and states of activation (naïve (N), early-activated (EA), cells with expression of IFN-stimulated genes (ISG), memory/exhausted precursors (M/Ex precursors), effector (Eff), effector/exhausted (Eff/Ex) and exhausted (Ex). **(D)** Representative canonical markers identifying T-cell subsets shown in **(C)** (see complete gene subset for each cluster in **Supplementary Table 2**). **(E)** Compositional analysis of different T-cell subsets by treatment. **(F)** Integrated trajectory analysis by Monocle-3 of the seven CD8^+^ T-cell subsets identified in **(C)**. White circle corresponds to the pre-established trajectory origin within the CD8^+^ TN. Gray circles correspond to the end point of the paths (P1-4). Black circles correspond to branch points of a given path.

Next, we conducted scRNA-seq studies to decipher transcriptomic networks determining the fate of drug-responsive CD8^+^ T-cells. These studies used LN cells from mice of all treatment groups at d4 of drug treatment. After sample and data processing, each cluster was annotated using a cell type marker-based approach (13). We identified 27 cell clusters corresponding to 10 different immune cell types (**Figure 5A**). Final annotations were verified by canonical marker expression (**Figure 5B**) (14–18). Subsequent sub-clustering (**Figure 5C**) and annotation of T-cells revealed the following subtypes (**Figure 5D**; **Supplementary Table 2**): a) naïve CD4^+^ and CD8^+^ T-cells (T_N_), with enriched expression of *Sell*, *Lef1* and/or *Tcf7*; b) early-activated CD8^+^ T-cells (T_EA_) with naïve and memory-like characteristics, expressing *Cd69* but not *Pdcd1*, possibly corresponding to PD1^-^CD69^+^CD25^-^ T-cells identified by FACS. Within the CD8^+^ T_EA_, a subpopulation showed high levels of IFN stimulated genes (ISG) including *Irf7* (T_N/EA_-_ISG_). T_EA_-1 expressed effector-related genes such as *Eomes, Cxcr3, Xcl1, Klrd1, Prf1, Cd28*, and *Gzmm* but also *Tcf7*. *Egr* genes, which are associated with T-cell anergy, characterized T_EA_-2; c) CD8^+^ T-memory and exhausted precursors cells (T_M+Ex precursors_) expressing *Pdcd1*, *Lag3*, *Cd28*, *Icos*, and *Nrp1*. The expression of early exhaustion transcription factors (*Tox* and *Batf) (* 17) but not *Cd69*, associated these cells with CD8^+^PD-1^+^CD69^-^ T-cells identified by FACS in DT+ABC mice at d4; d) Effector CD8^+^ T-cells (T_Eff/Ex_) and CD4^+^ T-cells (T_Eff_) were enriched in *Thy1, Spn, IL18r1, Cd7*, and integrin genes. CD8^+^ T_Eff/Ex_ cells also expressed *Mki67*, *Gzma* and *Gzmb*, *Pdcd1* and *Lag3*; e) CD8^+^ T_Ex_ showed similar markers of T_Eff/Ex_ but reduced *Mki67*, chemokine receptors, *Spn, Thy1*, *S100a6* and *Icos*; f) T_reg_ presented the highest levels of *Ctla4* and *Il2ra*, and *Foxp3*; and g) T-cells with *Tcr* gamma-delta genes (γδ-T).

As expected, T_N_ were dominant in Untr LN (**Figure 5E**). ABC LN had more CD8^+^ T_EA_ and T_N/EA-ISG_ than other treatment groups, as well as T_reg_. Treg ablation led to the accumulation of both CD8^+^ and CD4^+^ T-cells with effector or exhausted characteristics, particularly CD8^+^ T_Eff/Ex_ in DT+ABC LN (**Figure 5E**) in comparison to αCD4+ABC (**Supplementary Figure 8**). DT treatment did not achieve complete depletion of T_reg_, confirming FACS results (**Supplementary Figure 1D**).

The progression of the CD8^+^ T-cell response to drug was evaluated by pseudotime analysis of integrated data using Monocle 3 (19). Four key trajectories or “paths” were identified upon preestablishing the origin within CD8^+^ T_N_ (**Figure 5F**). Substantial differences were observed dependent on treatment (**Supplementary Figure 9**). Path 1, spanning mostly within T_N_, was predominant in Untr LN, in agreement with compositional analysis data (**Figure 5E**). Path 2 showed the progression of T_N_ to T_Eff/Ex_ through successively differentiated states (T_EA_-1, T_M/Ex precursors_). CD8^+^ T-cells in ABC LN were arrested at T_EA_-1 stage in path 2, while Treg ablation led LN CD8^+^ T-cells to evolve from T_EA_-1 to T_M/Ex precursors_ and further to T_Eff/Ex_, likely due to self-reactivity. However, CD8^+^ T_M/Ex precursors_ and T_Eff/Ex_ cells were found at higher frequency in DT+ABC LN possibly due to the enrichment of ABC-reactive CD8^+^ T-cells subsets in this group. Path 3 evolved from the split in path 2 towards T_EA_-2, present in all treatment groups. Path 4 connected T_N_ and T_N/EA-ISG_ with Path 2 at the level of T_M/Ex precursors_, and was mostly in cells of ABC and DT+ABC LN.

**Figure 6:**
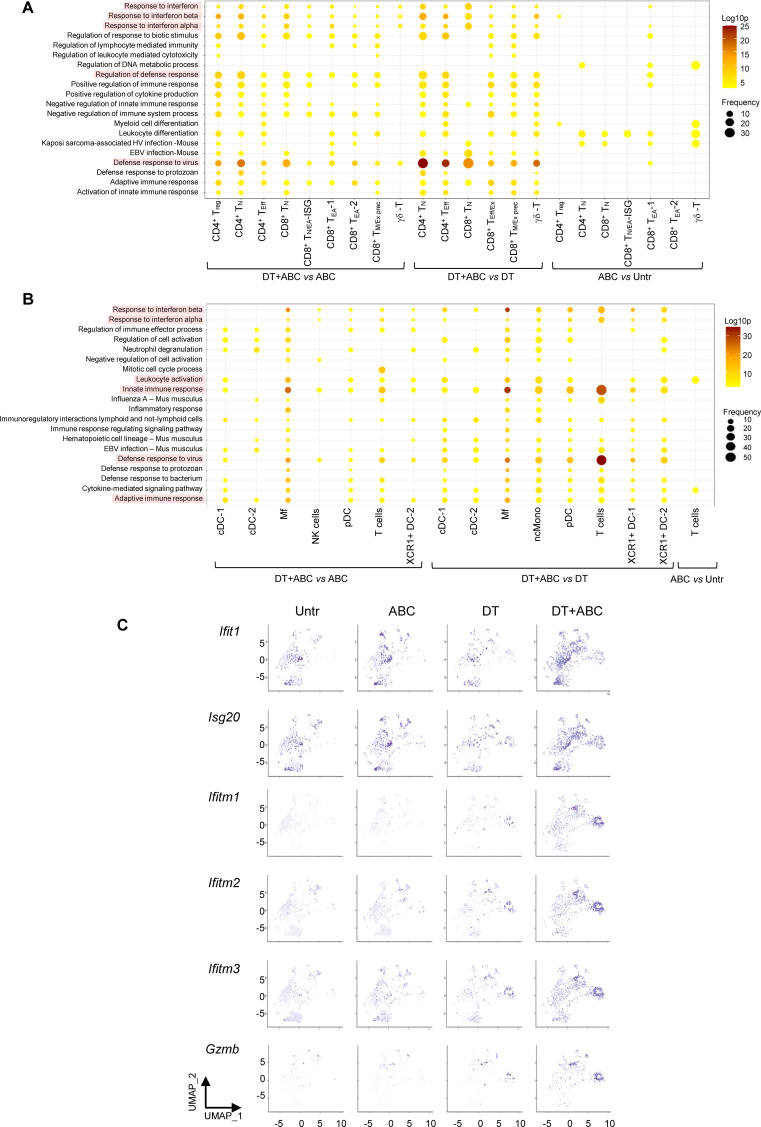
IFN signatures are over-represented in T-cells, Mf, pDC and ncMono from DT+ABC LN. **(A, B)** Over-representation analysis (ORA) of significantly differentially expressed genes (DEG) between DT+ABC and treatment controls (ABC or DT) within T-cell subsets **(A)** or the overall LN cell types **(B)** (see DEG list in **Supplementary Table 3**). ORA resulting from comparison of DEG in ABC vs Untr is also depicted for all cell types. **(C)** Feature plots showing the expression of highly represented DEG corresponding to several IFN-I and IFN-II stimulated genes and *Gzmb* in different T-cell subsets of animals treated with DT and/or ABC.

We conducted over-representation analysis (ORA) of significantly differentially expressed genes (DEG) in different cell subsets to identify associations between transcriptional networks and disease, comparing DT+ABC versus treatment controls (ABC or DT) and ABC versus untreated conditions. IFN/innate immune/viral defense pathways were identified as significantly enriched in T-cell subsets of DT+ABC LN (**Figure 6A**), in addition to macrophages, non-conventional monocytes, and pDC subsets (**Figure 6B**). Type-I IFN-related pathways were also present in CD8^+^ T_EA_-1 cells of ABC versus Untr LN.

As presented in **Supplementary Table 3**, IFN-I-associated genes (*Ifit1* and *Ifit3* among others), *Dusp1(a MAPK dual-specific phosphatase)*, and chemokine genes *Ccl5* and *Xcl1* that attract XCR1+ DCs were upregulated in ABC *vs* Untr LN, primarily in T_N_, T_EA_ and Treg. Genes of the activator protein 1 (AP-1) pathway were dominant in T-cells of the ABC LN, suggesting a potential role in the transient activation of drug-specific cells. In contrast, different T-cell subsets in DT+ABC LN showed upregulated DEG pertaining to different IFN networks. One group included IFN-I-stimulated genes *Ifit1, Ifit3, Isg20, Isg15, Rsad2 and Gbp*, in less differentiated T-cell subtypes (**Figure 6C**). These genes overlap with top canonical markers in T_N/EA-ISG._ The second group consisted of type IFN-I and II-stimulated genes *Ifitm1, Ifitm2 and Ifitm3* predominantly in CD8^+^ T_Eff/Ex_ and CD4^+^T_Eff_ cells (**Figure 6C**), but also in non-depleted Treg. Additional highly DEGs in DT+ABC *vs* ABC cells corresponded to effector molecules inducible by IFN-I, *Gzma, Gzmb, Eomes* (20), and by both IFN-I and IFN-II, such as chemokines and receptors (*Ccl2, Ccl5, Cxcr3*), cell cycle (*Mki67, Pclaf, Ube2c*), adhesion and motility (*Fscn1*), and anti-apoptotic (*Birc5*) genes. None of these IFN-I/IFN-II-related DEG were canonical markers for CD8^+^ T_N/EA-ISG_, suggesting their involvement in T-cell effector function.

**Figure 7:**
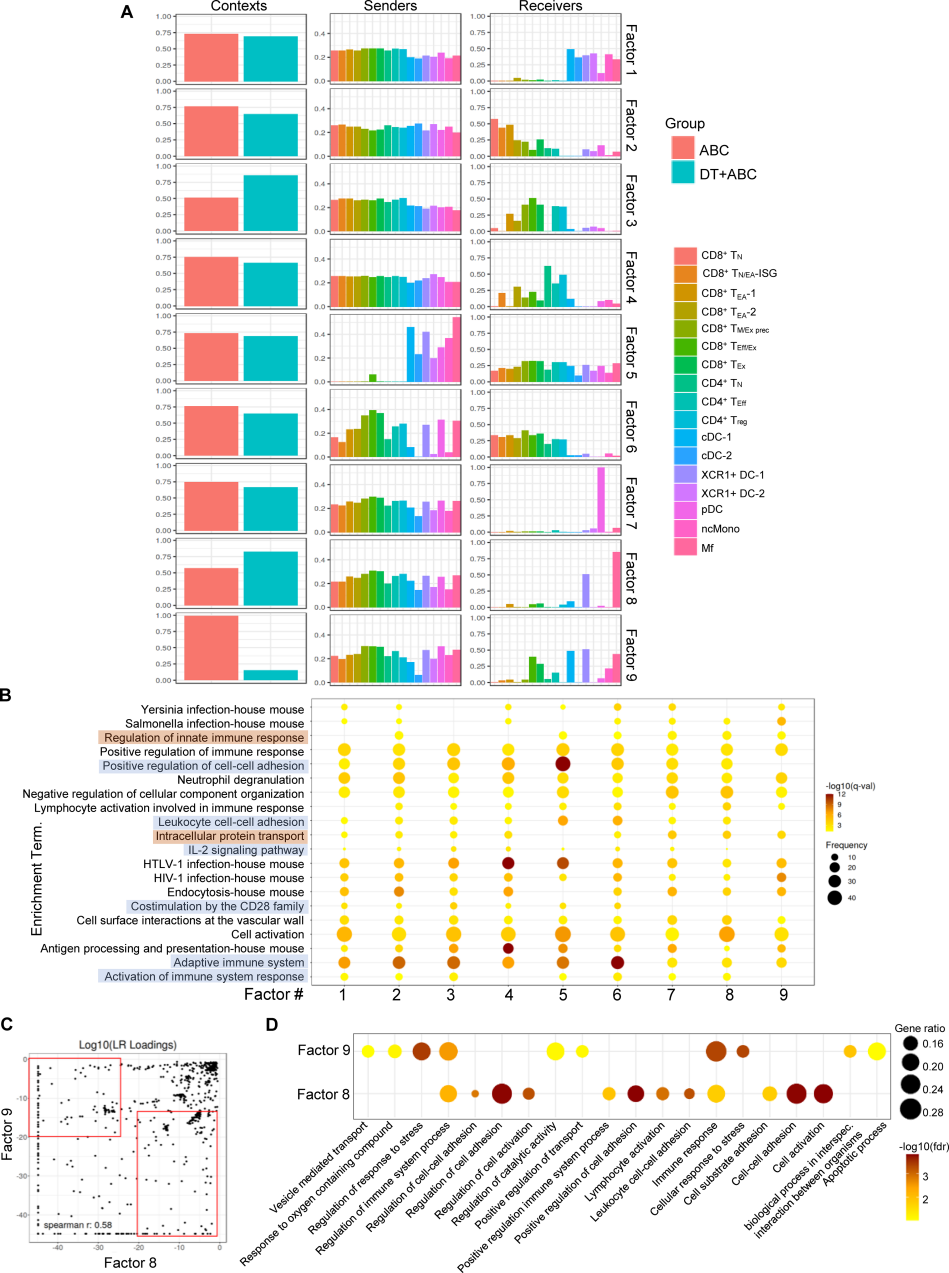
Cell-cell communication profiles reveal over-represented pathways associated with cellular stress early in ABC treatment. Ligand-receptor pair (LR) and sender-receiver analysis in LN cells of animals treated with ABC and DT+ABC using LIANA. **(A)** Context-dependent communication patterns (factors) between sender and receiver LN cells based on Tensor-cell2cell analysis. **(B)** ORA based on identified LR in **(A)** for the 9 different factors. Blue highlights represent pathways predominantly associated with factors 1–6 featuring adaptive immune responses. Brown highlights represent pathways predominantly associated with factor 7–9 and depicting innate immune responses. Data show frequency (dot size) and significance [false discovery rate (fdr)-adjusted p-value (q-value)]. **(C)** Comparison of LR loadings between factor 8 (predominant in DT+ABC context) and 9 (predominant in ABC context) [see **(A)**] with enriched LR pairs in each respective factor (red rectangles). **(D)** ORA based on factor-enriched LR loadings in **(C)**. Data show gene ratio (dot size) and significance (fdr).

Next, we performed cell-cell communication (CCC) analysis using a workflow that scores communication between ligand-receptor pairs (LR) and sender-receiver cell pairs using LIANA (21). Context-dependent communication patterns (or factors) were established using Tensor-cell2cell (22, 23). Out of nine identified factors, factors 3 was preferentially represented in DT+ABC LN (**Figure 7A**). All immune cell types (senders) communicated with receivers consisting of activated CD8^+^ and CD4^+^ T-cells implicating effector networks. In contrast, in factors 8 (predominant in DT+ABC) and 9 (mostly in ABC), senders were more terminally differentiated CD8^+^ and CD4^+^ T-cells as well as XCR1^+^ DC-1, pDC and Mf communicating mainly with XCR1^+^ DC-1 and Mf (receivers) in addition to cDC1 and CD8^+^ T_Eff/Ex_ in factor 9. Notably, factor 7 (similarly in both contexts) had pDC as the only receiver. ORA of genes associated with the top factor’s LRs segregated the 9 factors into two groups (**Figure 7B**). Adaptive immune CCC networks (highlighted in orange) characterized factor 3 and included IL-2 signaling, costimulation by CD28, and leukocyte adhesion. Factors 7, 8 and 9 were defined by innate immune regulation, intracellular protein transport, and lack of CD28 costimulation, representing CCC at earlier stages of the immune response (highlighted in blue). Comparison of LR between factors 8 and 9 identified high LR loadings in factor 8 (DT+ABC) corresponding to molecules related to adaptive immune networks involving adhesion, costimulation, chemokine/cytokines and respective receptors, and Wnt pathways (**Figure 7C**; **Supplementary Table 4**). Conversely, factor 9 (ABC) showed LR loadings such as *S100a*, *Mmp, Tgfb* and TNF-related genes which ORA linked to cell stress (perhaps oxidative stress-related), apoptosis, and intracellular transport networks (**Figure 7D**).

### ABC-driven T-cell activation and inflammation in DT+ABC mice are dependent on both IFN-I and IFN-II networks

**Figure 8:**
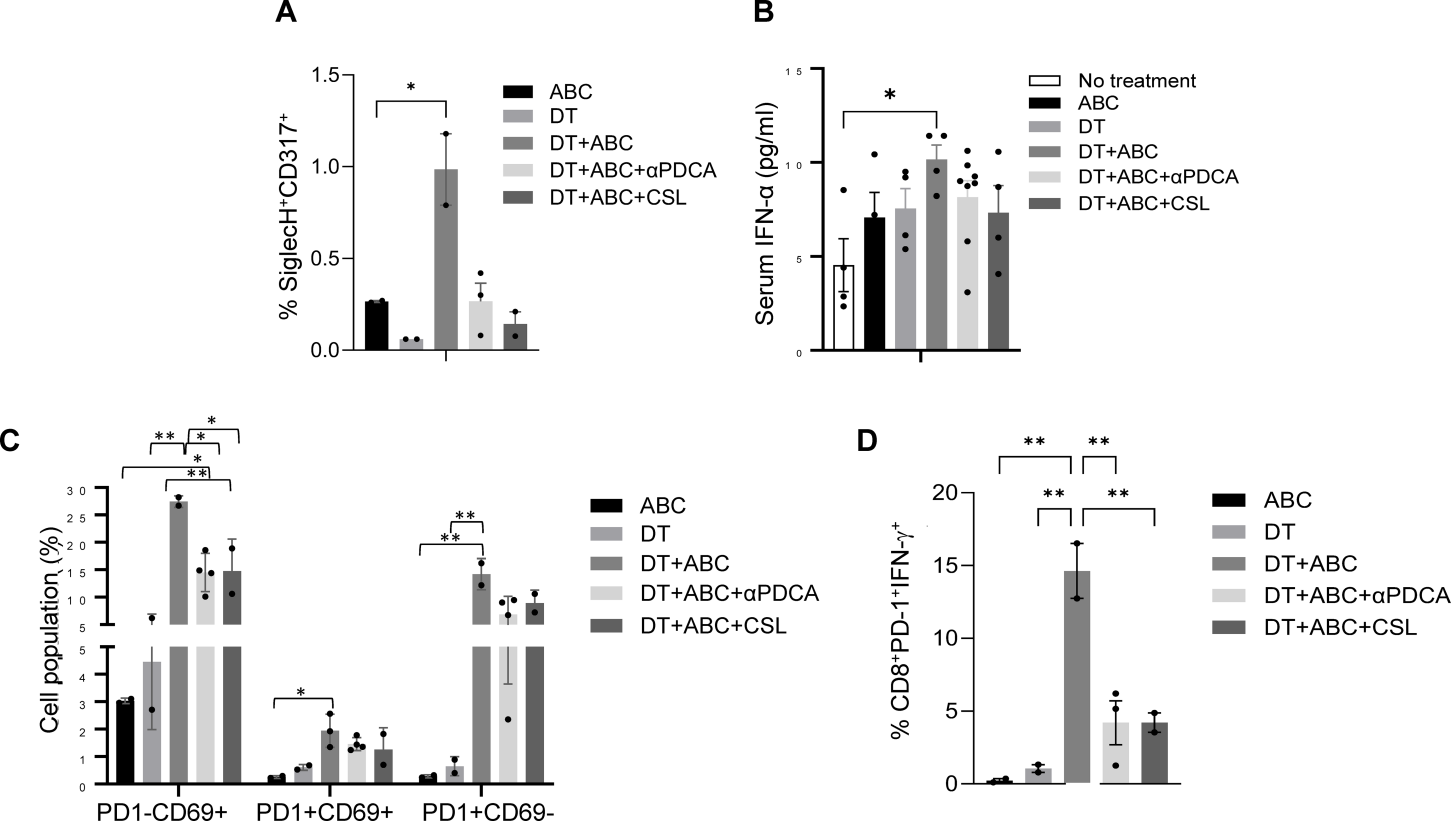
Macrophages and pDC are required for optimal CD8+ T-cell activation. Mice were treated with ABC and/or DT. A subset of DT+ABC mice were depleted of innate immune cell populations including pDC (with anti-PDCA antibody) or macrophages [with clodronate sulfate liposomes (CSL)]. Single cell suspensions were stained for FACS analysis at the end of treatment (day 8). **(A)** Percent of pDC (SiglecH+CD317+) in spleen. **(B)** Serum IFN-α levels by ELISA. **(C)** Percent of CD8^+^ T-cell subsets expressing PD1 and/or CD69 molecules in total splenocytes. **(D)** Percent of CD8^+^PD-1^+^ T-cells expressing IFN-γ upon *in vitro* restimulation of splenic cells of *in vivo* treated animals with 10 µg/mL of ABC (n = 2–3 mice/group). Statistics: one-way ANOVA; *P < 0.05 and **P < 0.01. Data are represented as mean ± SEM; individual dots correspond to different animals.

To confirm the role of IFNs in the activation and expansion of the immune response to ABC *in vivo*, we performed depletion experiments. Mice treated with DT+ABC showed an increase in splenic pDC and serum IFN-α, unlike animals that were depleted of pDC or Mf (**Figures 8A, B**). The lack of pDCs or Mf affected early-activated PD-1^-^CD69^+^ CD8^+^ T-cells as well as more mature CD8^+^ T-cell subsets expressing PD-1 (**Figures 8C, D**). PD-1^+^CD8^+^ T-cells failed to produce IFN-γ in response to ABC restimulation *in vitro* (**Figure 8D**).

**Figure 9:**
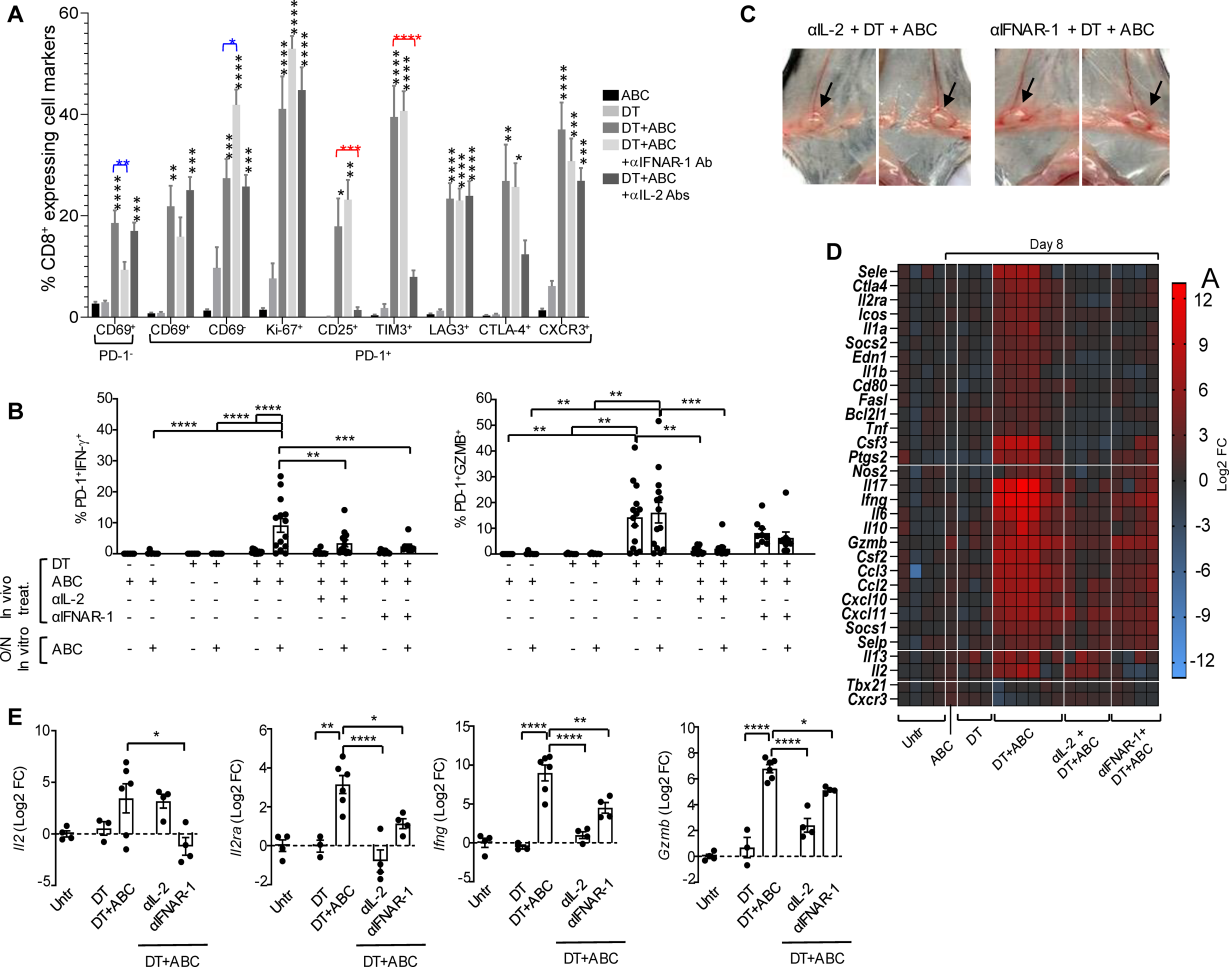
IFN-I and IL-2 signaling drive CD8^+^ T-cell activation by ABC in animals lacking Treg. Mice received treatment with ABC and/or DT. Additionally, some DT+ABC animals were treated with either anti-IFNAR1 mAb (to block IFN-I signaling) or a cocktail of mAb targeting IL-2 (to neutralize IL-2 signaling). Single cell suspensions were stained for FACS analysis at the end of treatment (day 8). **(A)** Percent of splenic CD8^+^ T-cell subsets expressing activation (CD69, CD25, CTLA4, CXCR3), immune checkpoint (PD-1, LAG3, TIM3), and proliferation (Ki-67) markers, in animals receiving different treatments. **(B)** Levels of activated CD8^+^PD-1^+^ T-cells upon *in vitro* cell restimulation with 10 µg/mL of ABC. **(C)** Lymphadenopathy (inguinal LN) after neutralization of IL-2 or blockade of IFN-I signaling in DT+ABC treated animals. See normal size LN in ABC animals in **Figure 1D** as reference. **(D)** Effect of IL-2 and IFN-I on RNA expression of immune-related genes in the spleen by real-time PCR. Data show mean log2 fold changes (FC) in treated animals as compared to the average of gene expression in untreated mice (n=3–6 mice/group). **(E)** Log2 FC in *Il2, Il2ra, Ifng and Gzmb* expression for individual animals depicted in **(D)**. Statistics: one-way ANOVA; *P < 0.05, **P < 0.005, ***P < 0.0005, and ****P < 0.0001.

In agreement with these results, blockade of IFNAR-1 reduced the accumulation of early-activated T-cells in DT+ABC mice but increased the presence of PD-1^+^CD69^-^ T-cells (**Figure 9A**) without affecting their high proliferation (Ki-67^+^) or expression of CD25 and terminally-differentiated markers. Drug-reactive cells, however, lost the ability to release IFN-γ and produced less GZMB when restimulated *in vitro* with the drug (**Figure 9B**). We also evaluated the effect of IL-2 for its role in proliferation/survival of T-cells and upregulating its receptor. IL-2 blockade abrogated the expression of CD25 and TIM3 in CD8^+^PD-1^+^ T-lymphocytes during DT+ABC treatment (**Figure 9A**) without significantly impacting their proliferation potential or expression of Lag3, CTLA-4 and CXCR3. Drug-reactive cells in DT+ABC animals deprived of IL-2 showed no ability to release IFN-γ or GZMB (**Figure 9B**).

Treatment of DT+ABC mice with αIFNAR-1 or αIL-2 Ab did not prevent lymphadenopathy (**Figure 9C**), in agreement with the high frequency of Ki67^+^ cells observed by FACS staining (**Figure 9A**). Notably, interfering with either cytokine pathway ameliorated the systemic inflammation resulting from DT+ABC treatment (**Figure 9D**). As shown in **Figure 9E**, blocking IL-2 abrogated the upregulation of *Il2ra* induced by DT+ABC treatment as well as the gene expression of downstream effector molecules, although IL-2 mRNA continued to be expressed. In contrast, IFN-I signaling was required to ensure the expression of *Il2*, and its blockade partially affected the transcription of *IL2ra* and effector molecules. Altogether these data indicate that IFN-α’s effect on T-cell differentiation occurs upstream of IL-2. Moreover, the effector function of drug-reactive cells require optimal IL-2 transcription and upregulation of CD25.

## Discussion

Drug hypersensitivity reactions linked to specific HLA alleles occur in low frequencies except in HLA-B*57:01^+^ patients treated with ABC. Studies in HLA Tg mice previously recreated ABC hypersensitivity reactions in models lacking CD4^+^ cells (6, 24) without conclusively demonstrating the role of Treg or additional host factors. Inflammatory triggers that induce innate immune activation, including those related to infections or drug cytotoxicity, may provide the appropriate environment for subsequent adaptive immune responses to develop. Nucleoside reverse transcriptase inhibitors such as ABC can reversibly terminate viral DNA synthesis but can also prevent mitochondrial DNA (mitDNA) strand elongation by binding mitDNA polymerase gamma (mitDNApol-g) with varying affinities (25). A drop in mitDNA levels impairs oxidative phosphorylation and promotes electron leakage resulting in oxidative damage compromising membrane integrity (26). MitDNA leakage into the cytoplasm activates cGAS/STING (27, 28) and/or TLR9 (29–31) pathways leading to expression of IFN-I and ISGs (32). *In vitro*, we showed that ABC was able to induce NF-kB activation and upregulation of *Ifna/b* and *Il1b* expression *in* murine RAW-Blue^TM^ macrophages. ABC synergized with a TLR7/8 agonist in causing mitochondrial dysfunction and caspase activity responsible for cellular stress and damage. Our data, consistent with results from ABC-treated human THP-1 cells (12), revealed ABC cytotoxicity in innate immune cells independently of HLA-B*57:01. Administration of ABC to Tg animals, however, was insufficient to break immune tolerance to the drug, suggesting the presence of additional host immune regulatory factors. Nevertheless, *in vivo* depletion of macrophages was later demonstrated to be key for the success of a drug-specific T-cell response (see further details later in the discussion), suggesting a possible role of innate immunity on ABC iDHR.

Treg can control autoimmune-like dysregulation (33–36), including suppressing ABC-induced CD8^+^ T-cell activation *in vitro* (**Figure 2B**). *In vivo*, Treg depletion with DT was necessary to enhance the development of ABC-driven effector CD8^+^ T-cell response as marked by PD-1 upregulation (37). Drug response in the absence of Treg was more rapid and robust than that observed in animals depleted of CD4^+^ cells (αCD4+ABC), suggesting a role for CD4^+^ non-Treg. Dermal infiltration of both CD8^+^ and CD4^+^ T-cells resembled that observed in skin biopsies of hypersensitive patients (38).

Dissecting the CD8^+^ T-cell response, we observed that in ABC mice, Tregs limited the expansion of early-activated CD8^+^ T-cells expressing CD69 and PD-1. This was likely mediated through interference with CD28/CD80/CD86 interactions between T-cells and drug-loaded DCs (39, 40) possibly through transendocytosis (41, 42), along with consumption of IL-2 (11, 43). In contrast, the absence of Treg in DT+ABC animals promoted CD8^+^ T-cell progression to more advanced functional states, reactivity to autocrine IL-2 and rapid response to drug restimulation *in vitro*. We believe that the source of IL-2 in our model comes mainly from CD8^+^ T-cells. Evidence supporting this reasoning is based on observed ABC reactivity in HLA Tg animals depleted of total CD4^+^ cells (**Supplementary Figures 4, 6**). In these animals, the drug induced robust CD8^+^ T-cell activation and function. We observed proliferating “self-reactive” CD8^+^ T-cells in DT mice and Tg^-^ DT+ABC animals which coexist with drug-specific cells in DT+ABC mice. Self-reactive T-cell expansion was dependent on CD28 and possibly on cytokine requirements other than IL-2 since the cells did not express CD25. These subpopulations may contribute to the accelerated CD8^+^ T-cell reactivity to drug observed in DT+ABC *vs* αCD4+ABC mice.

The development of a productive ABC response depends on antigen presentation and optimal costimulation by antigen presenting cells (6, 44). Removal of Treg led to rapid upregulation of CD80 and CD86 expression by DCs, resulting in activation of both autoreactive CD8^+^ and CD4^+^ T-cells through CD28 engagement along with TCR signaling, as seen in other models of Treg depletion (45). Following treatment with ABC, animals lacking Treg showed an increase of costimulatory molecules in both migratory and resident-like DC (data not shown). The role of Treg in suppressing multiple stages of T-cell activity was further demonstrated with blockade of DC costimulation by CTLA4-Ig in DT+ABC mice that prevented activation of all CD8^+^ T-cells while eliminating effector function and skin infiltration.

scRNAseq analysis of LN cells at d4 of ABC treatment revealed transcriptional networks determining the fate of T-cells. In ABC LN, early activated CD8^+^ T-cells (T_EA-1_) expressed *Fos/Jun* genes (AP-1) and *Dusp1*, IFN-I-stimulated genes and chemokines (*Ccl5* and *Xcl1).* AP-1 genes and *Dusp1* may contribute to T-cell anergy, triggered by incomplete T-cell receptor engagement or deficient CD28 signaling in the presence of Tregs. ISG expression on Tregs indicates IFN-I signaling, although not sufficient to impair the suppression function of these cells (46). Although CCL3 and CCL4 have been reported to attract IFN-I-producing pDC (47), in ABC-treated LN, CCL5 and XCL1 may function to recruit CCR5^+^ pDC, macrophages, and XCR-1+ DCs to DC/T-cell activation complexes (47).

In contrast, DT-treated mice (DT and to a greater extent DT+ABC animals) showed a higher percentage of CD8^+^ T-cell subsets expressing genes associated with effector, memory, and exhausted phenotypes (17, 48), as well as CD4^+^ T_Eff_ cells. Cell trajectory analysis showed, CD8^+^T_M/Exh precursors_ cells derived from CD8^+^T_EA_-1 or CD8^+^T_N_ rather than CD8^+^T_EA_-2 cells, which express anergy-related *Egr* genes. Subsequent progression of T_M/Exh precursors_ T_Eff_ and T_Ex_ phenotypes preferentially in DT+ABC LN confirmed FACS observations that both self and drug-reactive T-cells coexisted in these animals.

IFN-I signaling is a hallmark innate immune pathway that promotes DC function and CD8^+^ T-cell cross-priming and survival (49, 50). IFN-II signaling, largely restricted to NK cells, T-cells and DCs, is related to the development and expansion of adaptive immune responses. IFN downstream functions are exerted through the activation of ISG. In line with the possible role of ABC in inducing IFN, our study revealed a subset of cells, T_N/EA-ISG,_ with high expression of ISG mostly in LN of ABC and DT+ABC animals. Direct activation of CD8^+^ T-cells by IFN-I has also been reported in viral infections (50). Moreover, differentially expressed ISG related to IFN-I (containing ISRE elements) were predominantly found in CD8^+^T_EA_ cells from ABC LN but also in T_N_ and T_M/Exh precursors_ cells from DT+ABC LN. In contrast, IFN-I and IFN-II-stimulated genes such as *Ifitm*s (containing both GAS and ISRE elements) were enriched in both CD8^+^ T_Eff/Ex_ and CD4^+^T_Eff_ cells of DT+ABC (and perhaps at lower levels in DT) and thus associated with cells involved in adaptive immune response and self-reactivity. T-cells expressing IFN-I and IFN-II-stimulated genes have been reported in the skin of an ABC-reactive patient after a patch test (51). Innate immune cells, including macrophages, monocytes and pDC, which also displayed a strong DEG-ISG signature may affect immune-regulatory pathways (52–54). CCC studies revealed ligand-receptor pairs (LR) related to adaptive immune responses involving costimulatory and proliferative pathways (CD28, IL-2), mainly in DT+ABC LN. Conversely, factors with macrophages and pDC as receiver cells were associated with innate immune responses (factor 8 in DT+ABC LN and factor 9 in ABC LN). Notably, qualitative CCC differences were evident between these two factors reflecting transcriptomic programs that may determine the fate of drug-reactive cells in ABC-treated animals in the presence or absence of Treg. Oxidative stress and regulation of catalytic activity appeared as dominant transcriptomic signatures in ABC LN, consistent with drug-driven cytotoxic effects.

In this study we show that IFN-I, IL-2 and their respective receptors, IFNAR and CD25, play a role in the survival and effector function of drug-reactive T-cells. Systemic inflammation in DT+ABC mice was dampened by cytokine depletion unlike persistent lymphadenopathy induced by Treg ablation, indicating that the effect of these cytokines was on T-cell function rather than expansion. T-cell proliferation was responsible for LN enlargement, even when IL-2 signaling was impaired. This occurred through a CD28-driven IL-2-independent mechanism for both ABC-responsive CD8^+^PD-1^+^ and self-reactive CD8^+^PD-1^-^ T-cells, as well as for CD4^+^ non-Treg cells. Depletion of pDC, which produce high levels of type I IFN, and depletion of Mf, had similar effects on T-cell activation and function as blocking IFN-α signaling, suggesting that these cells may be the source of this cytokine. We determined that in the absence of Treg, IFN-α upregulates IL-2 gene transcription, which is required for the differentiation of drug-specific T-cells into effector cells. IL-2 production may result directly from IFN-α promoting expression of IL-2 and CD25 on T-cells (55); but also, indirectly from enhanced antigen presentation and TCR engagement. IL-2 signaling through CD25 needs to occur for IFN-γ production, contributing to antigen presentation (IFN-γ/IL-12 feedback loop) and effector T-cell response to drug. In contrast, while in ABC-treated animals similar pathways lead to the initiation of drug response, the presence of Treg interferes with APC maturation and IL-2 availability resulting in drug tolerance.

In summary, our data support a dual role of ABC in iDHR in activating both the innate and adaptive immune systems. ABC causes cell stress either directly or in combination with other factors, promoting innate immune cells to produce inflammatory molecules. In HIV patients taking ABC, virus infection may generate PAMPs/DAMPs that cooperatively enhance cytotoxicity caused by the drug, and lead to greater inflammation. Pro-inflammatory cytokines including IFN-I or IL-1β will promote DC maturation and chemokine-driven migration to the LN T-cell zone of ABC-treated mice. In these circumstances, DCs support early transient activation of CD8^+^ T-cells recognizing ABC-altered epitopes on HLA-B*57:01. However, T-cells subsequently undergo anergy or deletion if Treg are present to interfere with optimal costimulation through CD28 and/or availability of IL-2. To progress past this step, inactivation of Treg function is required to allow DC to support drug-reactive CD8^+^ T-cells, and recruitment of bystander help from self-reactive T-cells. Activated T-cells then produce IL-2, GZMB and IFN-γ which improve DC maturation and function, ensuring T-cell differentiation and effector function to exert systemic and cutaneous pathological effects.

The results of this study inform critical pathways that can be targeted for therapy, including the use of CTLA4-Ig to compensate for Treg malfunction by preventing CD28 engagement and T-cell activation. Cytokine inhibitors including monoclonal anti-cytokine and receptor antibodies and/or signaling blockers such as JAK inhibitors (56) would be useful to limit IFN-I, IFN-II and IL-2 T-cell expansion and effector function. In addition, small molecule inhibitors targeting granzymes, granulysin, and perforins could be included to attenuate tissue damage in drug reactions.

## Data Availability

Datasets are available on request: The raw data supporting the conclusions of this article will be made available by the authors, without undue reservation. All original code developed for the analysis of scRNAseq data has been deposited at https://doi.org/10.5281/zenodo.15532562.
