# Supplementary material for: Insights into regulatory T-cell and type-I interferon roles in determining abacavir-induced hypersensitivity or immune tolerance
Source: Front Immunol. 2025 Jun 6;16:1612451. doi: 10.3389/fimmu.2025.1612451 (PMC12178900; doi:10.3389/fimmu.2025.1612451)
Supplement: Supplementary file 1 [file DataSheet1.pdf]

SUPPLEMENTARY MATERIALS AND METHODS

RAW-Blue cell culture and analysis: Fifty-thousand RAW-Blue cells (InvivoGen) were cultured in DMEM containing 10% FBS,1% L-Glutamine and 1% PenStrep, (Invitrogen) for 16h in 96-well plates with 10 µg/ml Resiquimod (R848 (Invitrogen)), or 100 nM of CpG (FDA FBC Core, MD). After 8h incubation in TLR agonist-free medium, ABC was added for an additional 16h. NF-kB pathway activation was assessed by QuantiBlue (Invivogen) according to manufacturer's instructions. Changes in gene expression were evaluated as explained below. Similar cell culture conditions were used for mROS and cleaved Caspase-1 detection, except for a shorter (6h) incubation with ABC. Cells were co-stained *in situ* for 30 min with 2.5 µM MitoSOX (Thermo Fisher Scientific) and a 1% solution of FAM-coupled Z-YVADFmk (FAM-FLICA, Immunochemistry Technologies). Double-positive cells were identified by combined differential interference contrast and dual-channel fluorescence microscopy. Nikon NIS Elements AR 4.2 software was used for image overlays.

***In vivo* treatment:** DEREG HLA-B*57:01-transgenic mice were bred and housed under specific pathogen-free conditions in the AAALAC accredited animal facility of the U.S. FDA's Division of Veterinary Medicine (Silver Spring, MD) in compliance with the Institutional Animal Care and Use Committee regulations of the U.S. FDA (Protocol Number: #2017-11). Mice were treated with 1µg/mouse of DT (Sigma) at days -3, 0, 2 and 4 via intraperitoneal (*i.p*) injection. ABC dissolved in 30% DMSO (Ziagen tablets, GlaxoSmithKline) at 3 mg/dose was injected *i.p.* daily from day 0 to 4, and 7. The mouse equivalent dose of ABC was determined as described by Nair and Jacob(1). In addition, 0.2 mg of ABC in 70% sterile-filtered DMSO (Hybri-Max, SIGMA) was topically applied on each ear daily. Animals were sacrificed by day 8 unless otherwise specified. In certain experiments, Tg mice received 0.25 mg of α-CD4 mAb (clone: GK1.5, BioXcell) at days -3, 1, 4 and 7 of the experimental time course, CTLA-4-Ig (ORENCIA, abatacept), anti-IFNaR mAb (αIFNAR1, clone: MAR1-5A3, BioXcel) and anti-IL-2 mAbs (αIL-2, clones: JES6-1A12-and S4B6-1, BioXcell) at days 0, 3 and 6, *i.p*.

***Immunohistochemistry (IHC)*:** Ten percent formalin-fixed skin from the edge of the ear pinna was processed to paraffin blocks and sectioned (5 microns). H&E staining, and IHC (CD3ε (clone:

E4T1B, Cell signaling), CD4 (clone: RM4-5, Invitrogen) & CD8α (clone: 4SM15, Invitrogen)) were performed by Histoserv, Inc (Germantown, MD). Images were acquired using the Olympus VS-120 virtual microscope and processed in Adobe Photoshop.

Serum cytokines: Blood was collected at the time of euthanasia into Minicollect Z-serum Separator tubes (Greiner Bio-One, Germany) and spun to obtain serum. Serum was tested at 1:1 dilution (25 µL) using the Procarta Plex 36-plx kit (Thermo Fisher Scientific) and analyzed using a Luminex 100/200 instrument, as per manufacturer's recommendations. Alternatively, IFN-α was tested by ELISA (42115-1, PBL) using 50 µL of serum (1:1 dilution) and analyzed with a Spectra Max i3 reader (Molecular Devices).

***Flow cytometry*: ** Mice were phenotyped prior to the experiments to assess the expression of EGFP (under the FoxP3 promoter) and/or the HLA-B*57:01 molecule in circulating B-cells and T-lymphocytes (Antibody clone: B1.23.2, Invitrogen) using 50 µl of peripheral blood. Tg+ mice were identified based on the presence of the HLA-B*57:01 risk allele in B and T-cells, as well as the expression in blood $CD4^{+}$ T-lymphocytes of the DTR-EGFP fusion protein, which marked the $CD4^{+}$ Treg subpopulation (Tg+ ($EGFP^{+}HLA^{+}$) or Tg- ($EGFP^{+}HLA^{-}$)). Tregs are gated as live $CD3^{+}CD4^{+}$DTR-$EGFP^{+}$ ($Foxp3^{+}$) T-cells. Pretreatment of the samples for red blood cell lysis with ACK lysing buffer (GIBCO) and subsequent staining were performed following the method described in Cardone et al.(2). Intracellular staining was done using the BD Cytofix/Cytoperm Plus kit (BD Biosciences) as per manufacturer's instructions. Viability was assessed by LIVE/DEAD Fixable Aqua Dead Cell Stain Kit (Invitrogen) following manufacturer's directions while non-specific Ab binding was blocked with mouse Fc Block. After blocking, cells were stained for cell surface markers at 4°C for 30 min in the dark with 100 µl of a mixture constituted by 50% of Stain Buffer, 50% of Brilliant Stain Buffer (BD Horizon), and the appropriate diluted antibodies. Once labeled, cells were washed by centrifugation at 4°C for 5 min, fixed, permeabilized, and finally stained at 4°C for 30 min in the dark with anti-mouse IFN-g or GzmB mAb, or with anti-mouse CTLA-4 mAb. Once labeled, cells were washed twice with cold 1x BD Perm/Wash buffer (250 µl per sample) and finally resuspended in cold Stain Buffer prior to flow cytometry acquisition.

Antibodies used for cell surface marker staining were: anti-HLA B/C PE (clone B1.23.2, Invitrogen), anti-CD3 APC/Cy7 (clone: 17A2, Biolegend), anti-CD4 BV605 (clone: RM4-5, BD Horizon), anti-CD8a BV711 (clone: 53-6.7, BD Horizon), anti-CD45R (B220) APC (clone: RA3-6B2, Invitrogen), anti-CD19 Alexa Fluor 700 [clone: eBio1D3 (1D3), Invitrogen], anti-CD11b, BV605 (clone: M1/70, Biolegend), anti-CD11c APC (clone: N418, Biolegend), anti-Ly-6C BV785 (clone:HK1.4, Biolegend), anti-F4/80 Alexa700 (clone: BM8, Biolegend), anti-Siglec-H BV605 (clone: 551, BD Bioscience), anti-CD317 PE (clone: BST2, PDCA1, Biolegend), anti-CD62L Alexa Fluor 700 (clone: MEL-14, Biolegend), anti-CD44 PE (clone: IM7, Biolegend), anti-CD279 (PD-1) BV785 (clone: 29F.1A12, Biolegend), anti-CD223 (LAG-3) APC [clone: eBioC9B7W (C9B7W), Invitrogen], anti-CD366 (TIM3) PE-Cy7 (clone: RMT3 23, Invitrogen), anti-CD25 Alexa Fluor 488 (clone: eBio7D4 (7D4), eBioscience), anti-CD69 PeCy5 (clone: H1.2F3, Biolegend), anti-KLRG1 PE-Cy7 (clone: 2F1/KLRG1, Biolegend), anti-CD137 (4-1BB) APC (clone: 17B5, Invitrogen), anti-CD183 (CXCR3) APC & BV421 (clone: CXCR3-173, Biolegend), anti-I-Ab (Aβb) PE (clone: 25-9-17, Biolegend), anti-CD274 (B7-H1, PD-L1) BV421 (clone: 10F.9G2, Biolegend), anti-CD86 BV650 (clone: GL-1, Biolegend), and anti-CD80 PE-Cy5 (clone: 16- 10A1, Invitrogen). Antibodies used for intracellular staining were: anti-IFN-g APC (clone: XMG1.2, eBioscience), anti-Granzyme-B PE (clone: NGZB, Invitrogen), anti-CD152 (CTLA-4) APC (clone: UC10-4B9, Biolegend), and anti-Ki-67 PE (clone: 16A8, Biolegend). Flow cytometry data acquisition was performed using LSR Fortessa X20-SORP Cytek Aurora and analyzed by Diva 6.2/8.0.2 (BD Biosciences) and FlowJo (Version 10.10).

Single cell study: Prior to testing for scRNA-seq, samples were depleted of B cells (#130-121-301, Miltenyi), and enriched with Pan DCs (130-100-875, Miltenyi) to enhance detection of DC transcriptomes (except for Untr sample due to low levels of Pan DC recovered). A total of 10,000 LN cells per sample were used for the study, using a 10X Genomics Comptroller and the Chromium next GEM single cell 3' kit (v.3.1) (#1000130, 10X Genomics). Resulting libraries were sequenced with the Illumina NovaSeq SP kit. In all single cell RNA-seq analyses, default parameters were used unless otherwise stated.

a. Gene Expression Quantitation and Processing

Read alignment, unique molecular identifier (UMI) count quantification, and filtering of low-quality barcodes for each sample was performed with CellRanger's (version 6.1.1) *count* function(3). Mouse mm10 was used as reference. Raw UMI counts for each sample were processed and normalized using Seurat (v4.2.2)(4). First, cells with fewer than 200 non-zero genes and genes found in fewer than 3 cells were excluded from downstream analyses. To further limit the impact of low-quality cells(5), each sample was manually assessed with standard quality control metrics (the per cell total number of counts, total number of unique genes, and fraction of counts mapping to mitochondrial genes)(6) and filtered using heuristic thresholds as indicated in the table below(7).

TABLE: Heuristic thresholds used for sample QC in single-cell RNA-sequencing.

**Sample**	**Total UMI Counts**[a] [Lower, Upper]	**Total Unique Genes**[b] [Lower, Upper]	**Fraction of Counts that Mapped to Mitochondrial Genes**[c] [Upper]
Control	[3250, 20000]	[300, NA]	[12.5]
ABC	[2500, 25000]	[300, NA]	[12.5]
DT_ABC	[NA, 27500]	[NA, NA]	[10]
DT_Veh	[NA, 28000]	[NA, 6000]	[7.5]
aCD4_ABC	[NA, 27500]	[NA, NA]	[7.5]

a: Cells with UMI counts less than "Lower" or greater than "Upper" were excluded.

b: Cells with total unique genes less than "Lower" or greater than "Upper" were excluded.

c: Cells with a fraction of counts mapping to mitochondrial greater than "Upper" were excluded.

If a cell violates any of the three QC metric thresholds, it is excluded. NA indicates that a threshold was not applied.

Next, each sample was normalized for count depth and scaled by 10^6 to attain counts per million (CPM). We add a pseudo-count of 1 to the CPM values and transform them using natural log. Finally, we accounted for batch effects using Seurat's *IntegrateData*. First, for each sample, the top 2000 variable features for each normalized sample were identified using Seurat's *FindVariableFeatures* function to help identify integration features. The samples were then scaled (normalized by the standard deviation for each gene) and centered (subtracted the average expression for each gene) prior to running principal components analysis (PCA) to 50 principal components. Finally, the reciprocal PCA method was used to identify the integration anchors.

b. Dimensionality Reduction and Clustering

The integrated dataset was analyzed by PCA to reduce the dimensionality to 44 principal components (PCs), at which point the additional variance explained by considering more principal components was less than 0.1%. We ran the uniform manifold approximation and projection (UMAP) algorithm on the PCs to further reduce the dimensionality to two for visualization. We constructed a shared nearest neighbors (SNN) graph based on Euclidean distance in the PCA space with Seurat's *FindNeighbors* function. Next, we clustered cells using Louvain modularity optimization; the resolution parameter of Seurat's *FindClusters* function was set to 0.5. We visually confirmed that the integrated sample dataset did not possess batch-specific clusters; each cluster contained cells arising from multiple samples (data not shown).

c. Cell Type Annotation

We identified cell types for the cluster annotations by running ScType(8) on the scaled, integrated expression matrix. Input markers for each cell type were selected from the "Immune system" tissue of ScType's database. Clusters were annotated as the cell type that received the highest ScType score.

Clusters 12 and 13 were categorized by ScType as having low-confidence (a negative score or a score less than ¼ of the number of cells in that cluster). To verify cell type annotations for these and all other clusters, a list of markers for each cluster was identified using differential expression (DE) analysis. We tested for significantly up-regulated genes in a given cluster relative to all other cells in the dataset using a Wilcoxon rank-sum test. The log-normalized counts were used as input, and only genes present in at least 25% of cells in the cluster and that showed at least a $\log_2$-fold-change (LFC) of 0.5 were tested. Genes with Bonferroni corrected p-values less than or equal to 0.1 and a LFC greater than 0.75 were retained for assessment. Based on these marker outputs, we replaced a few of the ScType annotations: we re-annotated cluster 5 from basophils to conventional dendritic cells, cluster 13 from natural killer cells to $Xcr1^+$ dendritic cells, and cluster 16 from progenitor cells to $Xcr1^+$ dendritic cells. Due to a lack of markers, we maintained cluster 12 as an "unknown" cell type.

We also identified and labeled “sub-clusters” within subsets of the data to gain a higher resolution cell type annotation. For sub-clustering and annotation, we subsetted the batch-corrected expression matrix to cells only in a given cluster and re-ran the dimensionality reduction and clustering as previously described. The dimensionality for sub-clustering was set to 30 PCs. Marker genes for each subcluster were identified using the same DE test and criteria as described above, with the exception that the LFC threshold was set to 0.5 rather than 0.75. Cell types were manually annotated based on significantly differentially expressed genes within these sub-clusters. We refer to sub-clusters as a “Level 2” annotation, and the original clusters as a “Level 1” annotation. Level 2 cluster IDs were continuously numbered starting at one above the highest number in the Level 1 annotation. Thus, since Level 1 cluster IDs were annotated from 0-26, Level 2 annotations began at 27.

Due to uncertainty in the markers identified for Level 1 cluster 22, which ScType annotates as plasmacytoid dendritic cells, we further sub-cluster these cells, identifying three sub-clusters (Level 2 clusters 27-29). Cluster 27 was re-labeled as a conventional dendritic cell, and 28-29 were labeled as macrophages. To achieve higher granularity in T-cell annotation, we also applied this sub-clustering approach simultaneously to all ScType clusters annotated as T-cells (Level 1 clusters 0, 2, 3, 6, 10, 15, 21, and 24). From these 8 clusters, we achieved a total of 16 sub-clusters (Level 2 clusters 30-45) that we manually annotated (Supplementary Table 2).

d. Differential Expression Analysis

Differential expression (DE) analysis between the same cell types across different samples was conducted using MAST (v1.24.0)(9). DE was performed on the log-normalized expression matrix. To account for technical variability and other nuisance factors, we introduced the cellular detection rate (CDR), the fraction of genes expressed in a cell--as a covariate in the design matrix. We tested for significant differential expression using a likelihood ratio test; the full model regressed expression on both the test condition and the CDR, and the reduced model regressed expression only on the CDR. For each comparison, we only tested genes present in at least 10% of cells in either condition and that showed a LFC of at least 0.5. Genes with a Benjamini-Hochberg false discovery rate (FDR) less than or equal to 0.1 were considered significantly differentially expressed. Finally, we ran an over-representation analysis (ORA) of significantly differentially

expressed genes across all results simultaneously using Metascape(10), with all genes in the dataset as the background.

e. Trajectory and Cell-Cell Communication Analysis

Trajectory analysis of $CD8^+$ T-cells was performed using Monocle 3 (*v1.3.1*)(11). Applying Monocle 3 to the UMAP embedding identified from the T-cell sub-clusters (see *Cell Type Annotation* section for details), we apply the trajectory analysis workflow as described cluster cells using Leiden community detection, learn the principal trajectory graph, and order cells according to their pseudotime. For this final step, we select the trajectory graph root node as that which contains the largest fraction of cells that come from those annotated as $CD8^+$ Naive T-cells.

Cell-cell communication (CCC) analysis was conducted by applying a workflow(12) that combines LIANA(13) and Tensor-cell2cell(14). LIANA (v0.1.12) was used to calculate a communication score between cell type pairs for each sample, using the log-normalized gene expression matrix and LIANA's default consensus ligand-receptor interaction prior knowledge resource as inputs. We used the consensus magnitude rank score as the final communication score and subtracted this score from 1 to weigh scores more highly according to their relative importance. Next, we identified context-dependent CCC patterns by using Tensor-cell2cell (cell2cell v0.6.5) to build and decompose a 4D-tensor of these communication scores, with the samples set as the context dimension. We excluded ligand-receptor pairs that were not present in at least ⅓ of samples. For the decomposition, we mask ligand-receptor pairs that are not present in a given sample and set communication scores for cell types that are not present in a given sample to 0.

ORA was run simultaneously on all identified factors with Metascape(10) , using the top 10% of LR pairs by loading for each factor as input and setting all genes in the dataset that are present in the consensus resource as the background. A separate ORA was conducted on LR pairs specific to either factor 8 or factor 9. To do so, first, factor-specific LRs were identified as those that had high loadings in one factor and low loadings in the other. Specifically, Factor-8-specific LRs were taken as those with $\log_{10}$(loading) $\geq$ -20 in Factor 8 and $\log_{10}$(loading) $\leq$ –15 in Factor 9. Factor-8-specific LRs were taken as those with $\log_{10}$(loading) $\geq$ -20 in Factor 9 and $\log_{10}$(loading) $\leq$ –25 in Factor 8. Cluster Profiler(15) (v4.7.1.003) was used for enrichment, with the database mapping LR pairs from the consensus resource to the Gene Ontology biological processes being generated using cell2cell as previously described(14)). A Benjamini-Hochberg multiple test correction was

applied to the p-values for each pathway, and significant ($q \leq 0.1$) pathways were subsequently rank ordered by first their gene ratio, then their false discovery rate (FDR), and finally their gene count.

REFERENCES

1. Nair AB, Jacob S. A simple practice guide for dose conversion between animals and human. J Basic Clin Pharm. 2016;7(2):27-31.
2. Cardone M, Garcia K, Tilahun ME, Boyd LF, Gebreyohannes S, Yano M, et al. A transgenic mouse model for HLA-B*57:01–linked abacavir drug tolerance and reactivity. The Journal of Clinical Investigation. 2018;128(7):2819-32.
3. Zheng GX, Terry JM, Belgrader P, Ryvkin P, Bent ZW, Wilson R, et al. Massively parallel digital transcriptional profiling of single cells. Nat Commun. 2017;8:14049.
4. Hao Y, Hao S, Andersen-Nissen E, Mauck WM, 3rd, Zheng S, Butler A, et al. Integrated analysis of multimodal single-cell data. Cell. 2021;184(13):3573-87.e29.
5. Ilicic T, Kim JK, Kolodziejczyk AA, Bagger FO, McCarthy DJ, Marioni JC, et al. Classification of low quality cells from single-cell RNA-seq data. Genome Biology. 2016;17(1):29.
6. Luecken MD, Theis FJ. Current best practices in single-cell RNA-seq analysis: a tutorial. Molecular Systems Biology. 2019;15(6):e8746.
7. Mathys H, Davila-Velderrain J, Peng Z, Gao F, Mohammadi S, Young JZ, et al. Single-cell transcriptomic analysis of Alzheimer's disease. Nature. 2019;570(7761):332-7.
8. Ianevski A, Giri AK, Aittokallio T. Fully-automated and ultra-fast cell-type identification using specific marker combinations from single-cell transcriptomic data. Nature Communications. 2022;13(1):1246.
9. Finak G, McDavid A, Yajima M, Deng J, Gersuk V, Shalek AK, et al. MAST: a flexible statistical framework for assessing transcriptional changes and characterizing heterogeneity in single-cell RNA sequencing data. Genome Biol. 2015;16:278.
10. Zhou Y, Zhou B, Pache L, Chang M, Khodabakhshi AH, Tanaseichuk O, et al. Metascape provides a biologist-oriented resource for the analysis of systems-level datasets. Nature Communications. 2019;10(1):1523.
11. Cao J, Spielmann M, Qiu X, Huang X, Ibrahim DM, Hill AJ, et al. The single-cell transcriptional landscape of mammalian organogenesis. Nature. 2019;566(7745):496-502.
12. Baghdassarian HM, Dimitrov D, Armingol E, Saez-Rodriguez J, Lewis NE. Combining LIANA and Tensor-cell2cell to decipher cell-cell communication across multiple samples. Cell Reports Methods. 2024;4(4):100758.
13. Dimitrov D, Türei D, Garrido-Rodriguez M, Burmedi PL, Nagai JS, Boys C, et al. Comparison of methods and resources for cell-cell communication inference from single-cell RNA-Seq data. Nature Communications. 2022;13(1):3224.
14. Armingol E, Baghdassarian HM, Martino C, Perez-Lopez A, Aamodt C, Knight R, et al. Context-aware deconvolution of cell–cell communication with Tensor-cell2cell. Nature Communications. 2022;13(1):3665.
15. Wu T, Hu E, Xu S, Chen M, Guo P, Dai Z, et al. clusterProfiler 4.0: A universal enrichment tool for interpreting omics data. Innovation (Camb). 2021;2(3):100141.
