# Supplementary material for: Insights into regulatory T-cell and type-I interferon roles in determining abacavir-induced hypersensitivity or immune tolerance
Source: Front Immunol. 2025 Jun 6;16:1612451. doi: 10.3389/fimmu.2025.1612451 (PMC12178900; doi:10.3389/fimmu.2025.1612451)
Supplement: Supplementary file 2 [file Image1.pdf]

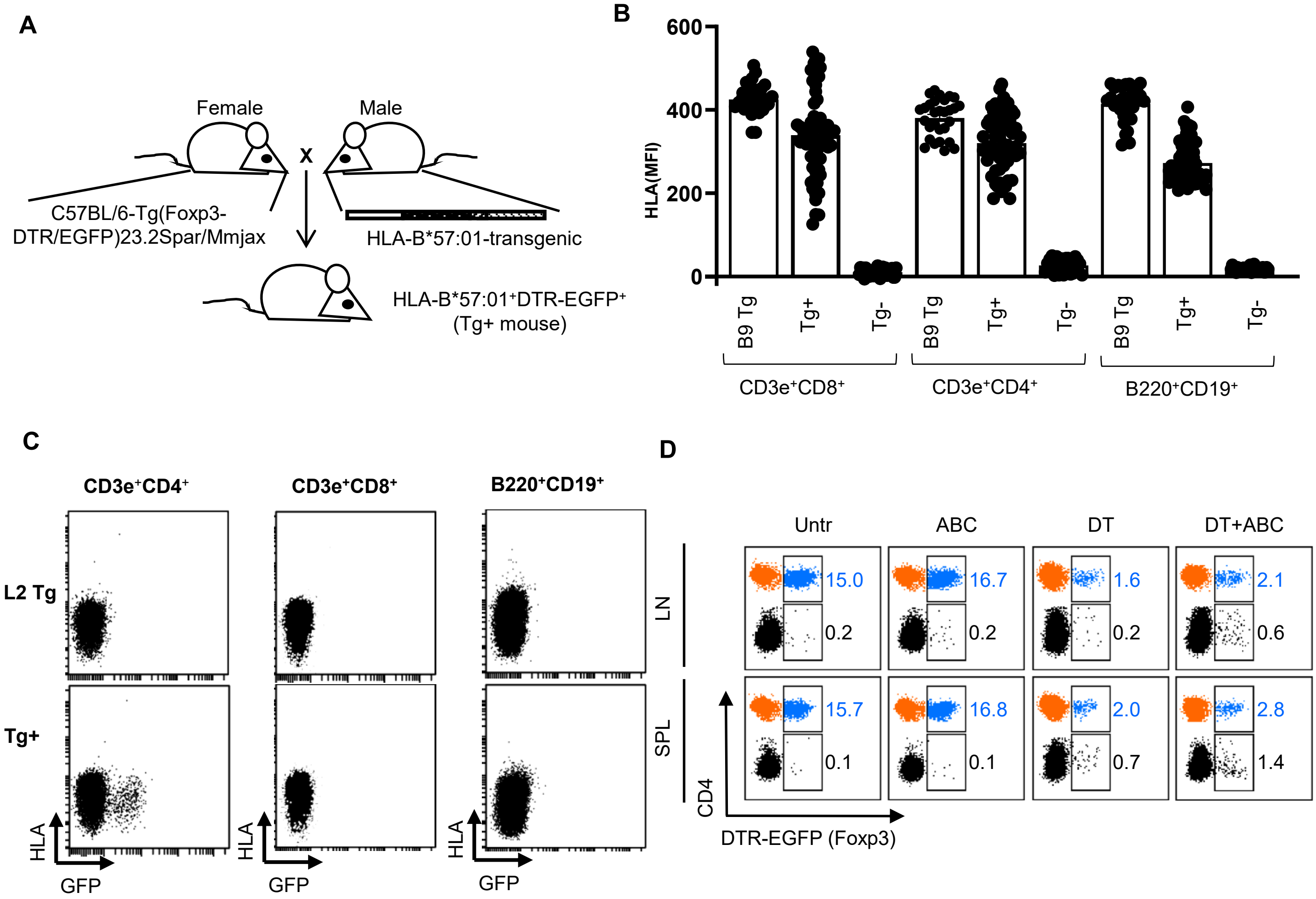

Supplementary Figure 1: Generation and phenotype of HLA-B*57:01^{+}DTR-EGFP^{+} mice and effect of DT on Treg levels.

(A) Generation of HLA-B*57:01^{+}DTR-EGFP^{+} mice (hereafter referred as "mice") by crossing hemizygous C57BL/6-Tg (Foxp3-DTR/EGFP)23.2Spar/Mmjax female mice with HLA-B*57:01-transgenic male animals. (B) Expression of the HLA-B*57:01 transgene in T (CD3e^{+}CD8^{+} and CD3e^{+}CD4^{+}) and B (B220^{+}CD19^{+}) lymphocytes from different mouse strains. (C) Flow cytometry strategy to identify mice carrying HLA-B*57:01 risk allele in blood B- and T-cells, and expressed the DTR-EGFP fusion protein in blood CD4^{+} T-cells (Tg+), as opposed to L2 Tg animals that lack GFP expression. (D) Representative flow cytometry dot plot of levels of Treg (gated as live CD3^{+}CD4^{+}DTR-EGFP^{+}(Foxp3^{+}) T-cells) in the spleen (SPL) and lymph node (LN) of mice left Untr, or treated with ABC, DT or a combination of DT+ABC.

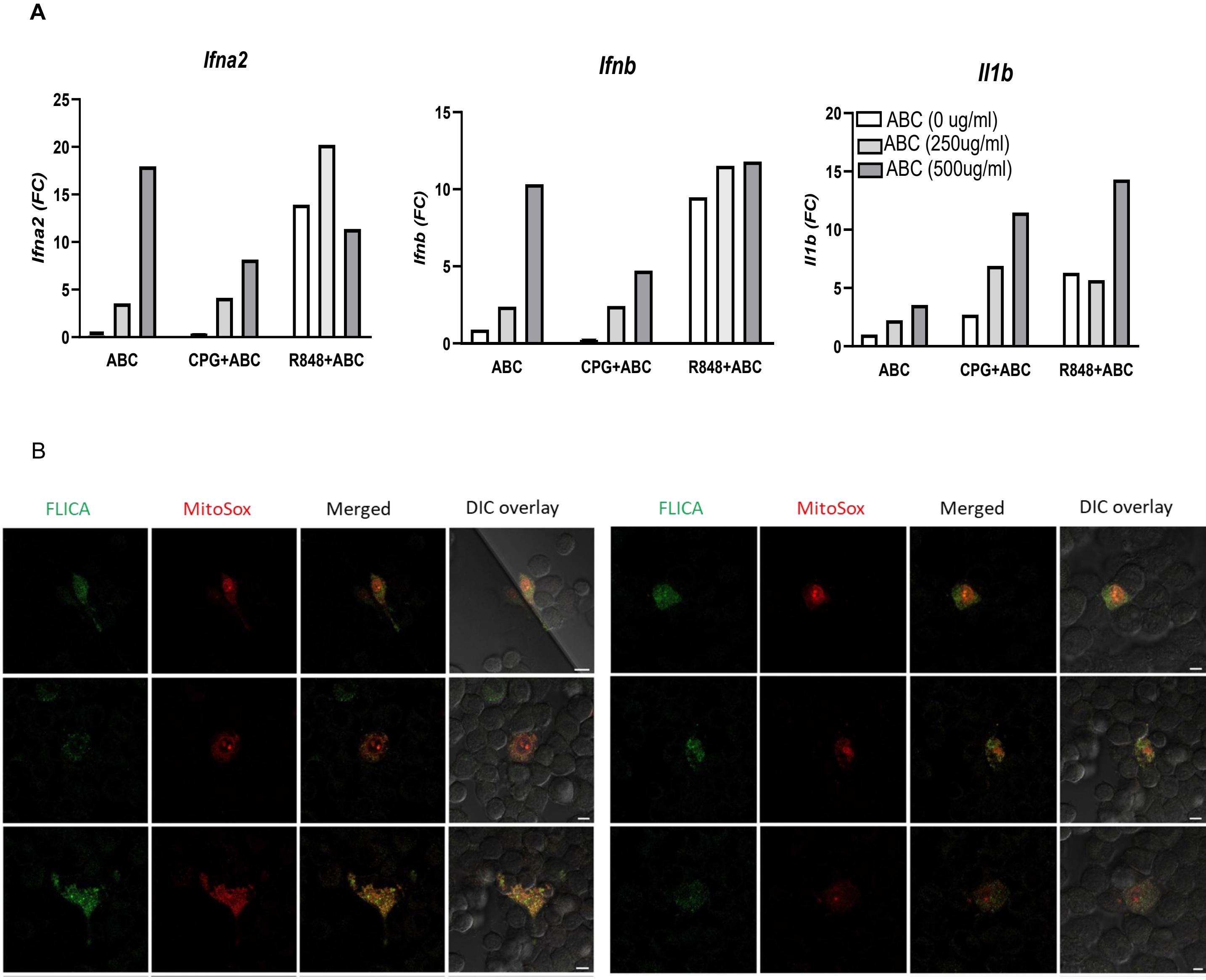

Supplementary Figure 2: Supporting information for ABC induction of innate immune activation through cellular stress pathways. Murine macrophage RAW-Blue cells were cultured with drug and TLR agonists as indicated in materials and methods. (A) IFN-I and IL-1B gene expression was measured by Taqman as in Figure 2B. This is a representative independent experiment out of two with comparable results. (B) RAW-Blue cells were cultured in the presence of ABC (250µg/ml) and/or R848 (10µg/ml) as indicated in materials and methods. Cells were evaluated for caspase activity (FAM-FLICA) and ROS production (MitoSOX) by immunofluorescent methods. Double-positive cells were identified using combined differential interference contrast and dual-channel fluorescence microscopy (Scale bar-10 µm). Panels across show 6 independent microscopic fields.

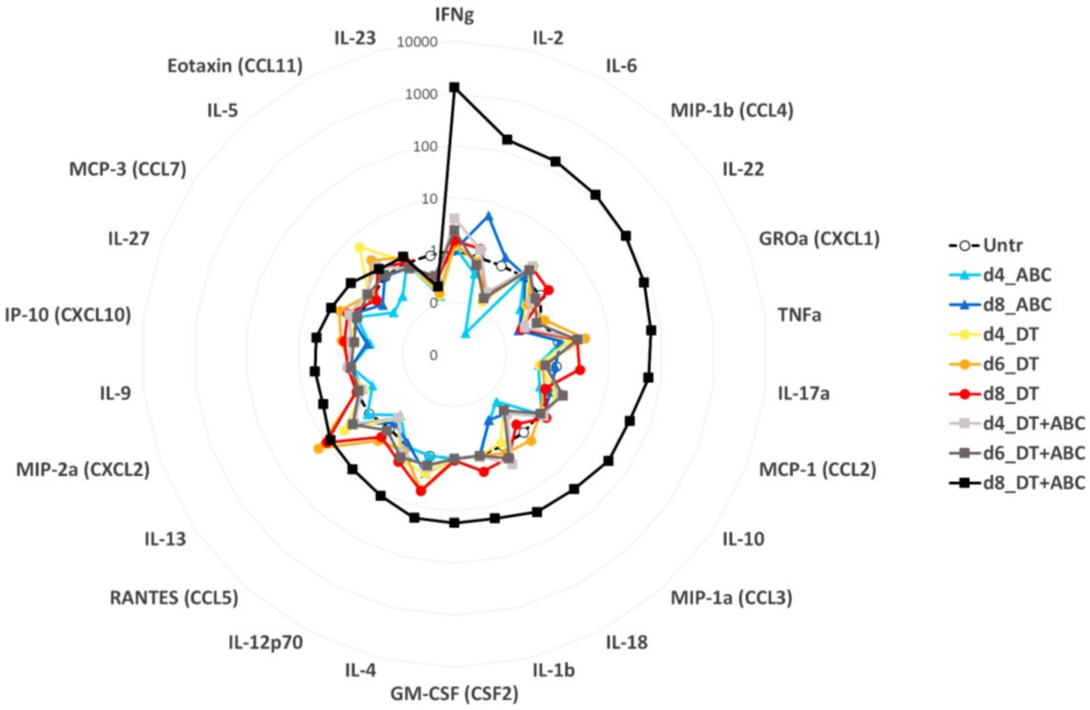

Supplementary Figure 3: DT+ABC mice experience systemic cytokine storm by day 8 of treatment. Mice were treated with ABC, DT or a combination of DT+ABC as indicated in materials and methods**.** Cytokine and chemokine analysis of serum samples at the end of treatment using multiplex array. Radial plot shows fold increase in the serum concentration of the different parameters between treated mice and untreated (Untr) animals. Data points represent geometric mean values from 2-9 mice/group.

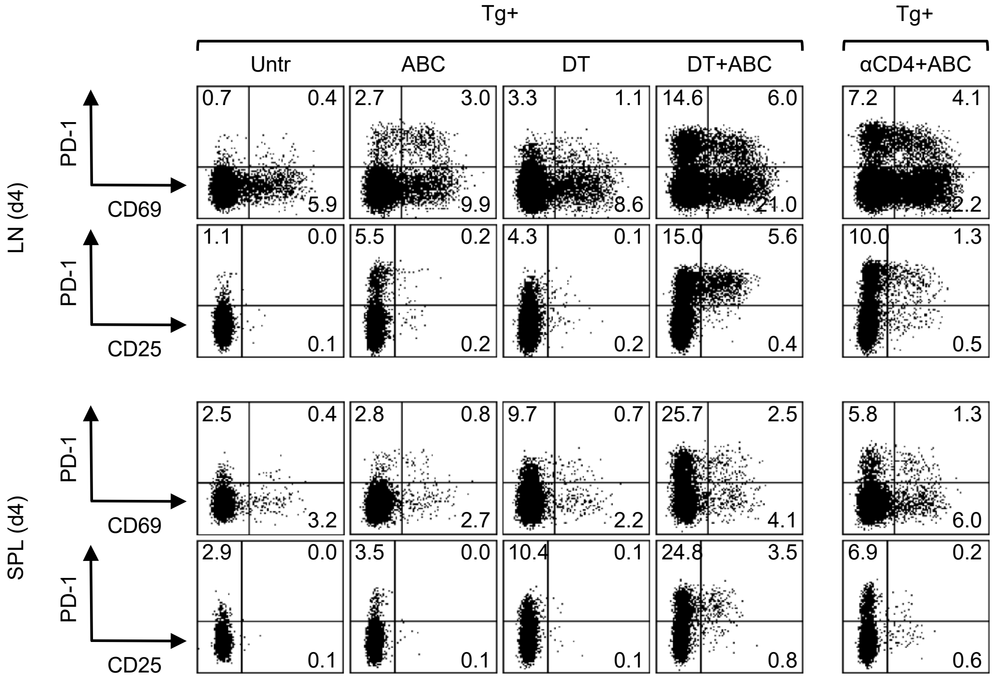

Supplementary Figure 4: Systemic activation and accumulation of $CD8^+$ T-cells in mice treated with ABC, and/or depleted of Treg, and total $CD4^+$ cells. Animals were treated with ABC, DT, DT+ABC or αCD4 Ab+ABC as indicated in supplementary methods. Spleen (SPL) or lymph nodes (LN) were collected at day 4 of treatment. Representative flow cytometry dot plot with the expression level of activation markers ($CD69^+$ and $CD25^+$) on $PD\text{-}1^+CD8^+$ T-cells. The experiment was conducted with 4-8 mice/group.

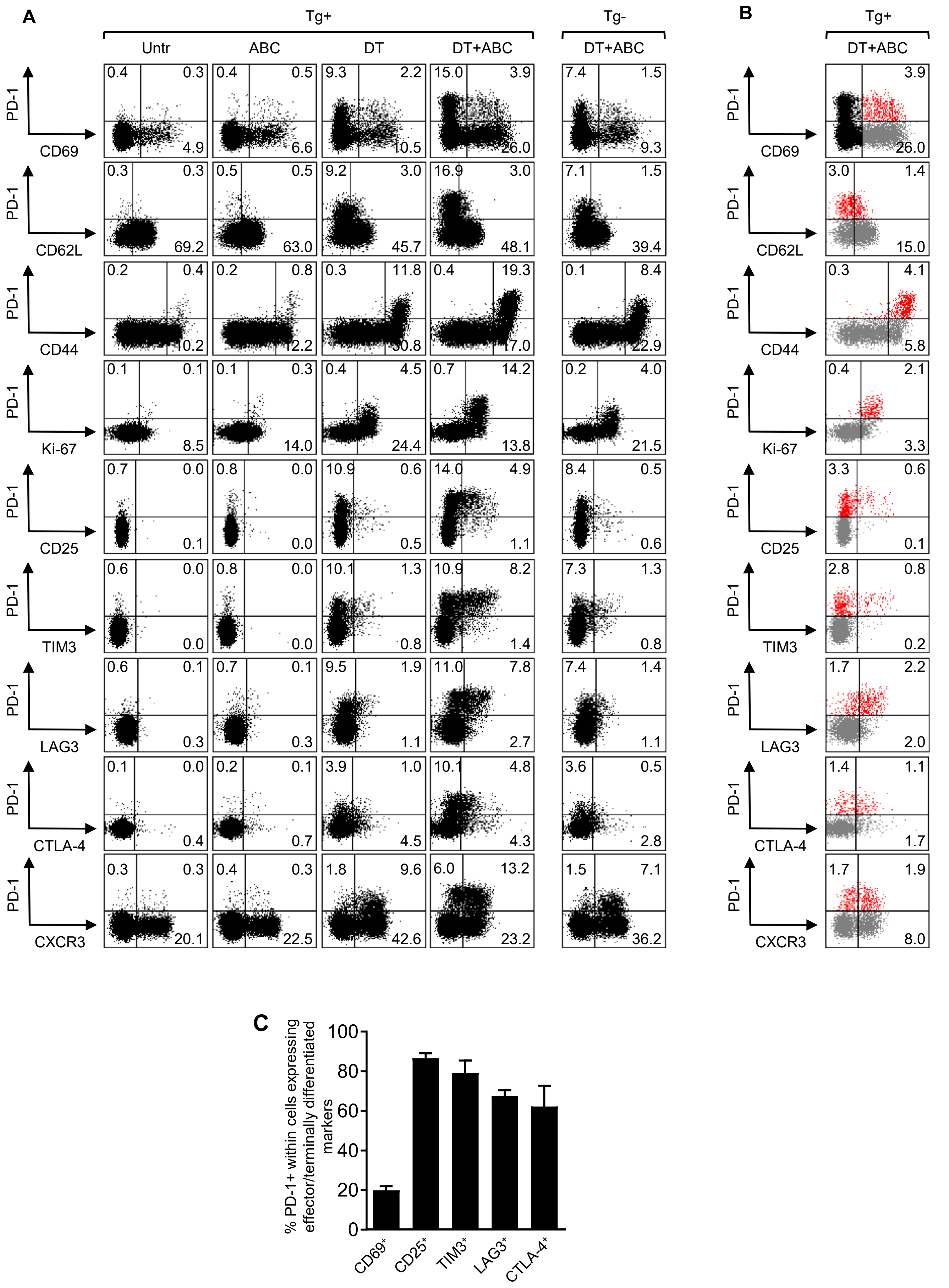

A
Tg+
Untr
ABC
DT
DT+ABC
Tg-
DT+ABC
B
Tg+
DT+ABC
PD-1
CD69
CD62L
CD44
Ki-67
CD25
TIM3
LAG3
CTLA-4
CXCR3
0.4 0.3 4.9
0.4 0.5 6.6
9.3 2.2 10.5
15.0 3.9 26.0
7.4 1.5 9.3
3.9 26.0
0.3 0.3 69.2
0.5 0.5 63.0
9.2 3.0 45.7
16.9 3.0 48.1
7.1 1.5 39.4
3.0 1.4 15.0
0.2 0.4 10.2
0.2 0.8 12.2
0.3 11.8 30.8
0.4 19.3 17.0
0.1 8.4 22.9
0.3 4.1 5.8
0.1 0.1 8.5
0.1 0.3 14.0
0.4 4.5 24.4
0.7 14.2 13.8
0.2 4.0 21.5
0.4 2.1 3.3
0.7 0.0 0.1
0.8 0.0 0.0
10.9 0.6 0.5
14.0 4.9 1.1
8.4 0.5 0.6
3.3 0.6 0.1
0.6 0.0 0.0
0.8 0.0 0.0
10.1 1.3 0.8
10.9 8.2 1.4
7.3 1.3 0.8
2.8 0.8 0.2
0.6 0.1 0.3
0.7 0.1 0.3
9.5 1.9 1.1
11.0 7.8 2.7
7.4 1.4 1.1
1.7 2.2 2.0
0.1 0.0 0.4
0.2 0.1 0.7
3.9 1.0 4.5
10.1 4.8 4.3
3.6 0.5 2.8
1.4 1.1 1.7
0.3 0.3 20.1
0.4 0.3 22.5
1.8 9.6 42.6
6.0 13.2 23.2
1.5 7.1 36.2
1.7 1.9 8.0
C
% PD-1+ within cells expressing effector/terminally differentiated markers
0
20
40
60
80
100
CD69+
CD25+
TIM3+
LAG3+
CTLA-4+

Supplementary Figure 5: Activation phenotype of PD-1 and CD69-expressing $CD8^+$ T-cells in lymph nodes at day 8 of treatment. Tg+ or Tg- mice were treated with ABC, DT, or DT+ABC, or left untreated (Untr) as per supplementary methods. Lymph nodes were collected at the end of treatment and single cell suspensions were subsequently analyzed by FACS (n=5 mice/group). (A) Representative flow cytometry dot plot with expression levels of activation markers (CD69, CD62L, CD44, CD25, CXCR3), proliferation marker (Ki-67^+), and terminal differentiation markers (TIM3, LAG3, CTLA-4) on $CD8^+$ T-cells. (B) Representative flow cytometry dot plot with the expression levels of activation, proliferation, and terminal differentiation markers on $CD8^+PD\text{-}1^+CD69^+$ (red) and $CD8^+PD\text{-}1^-CD69^+$ (gray) T-cells of DT+ABC-treated Tg+ mice. (C) Summary of levels of expression of PD-1^+ in cells expressing effector or terminally differentiated markers from (B).

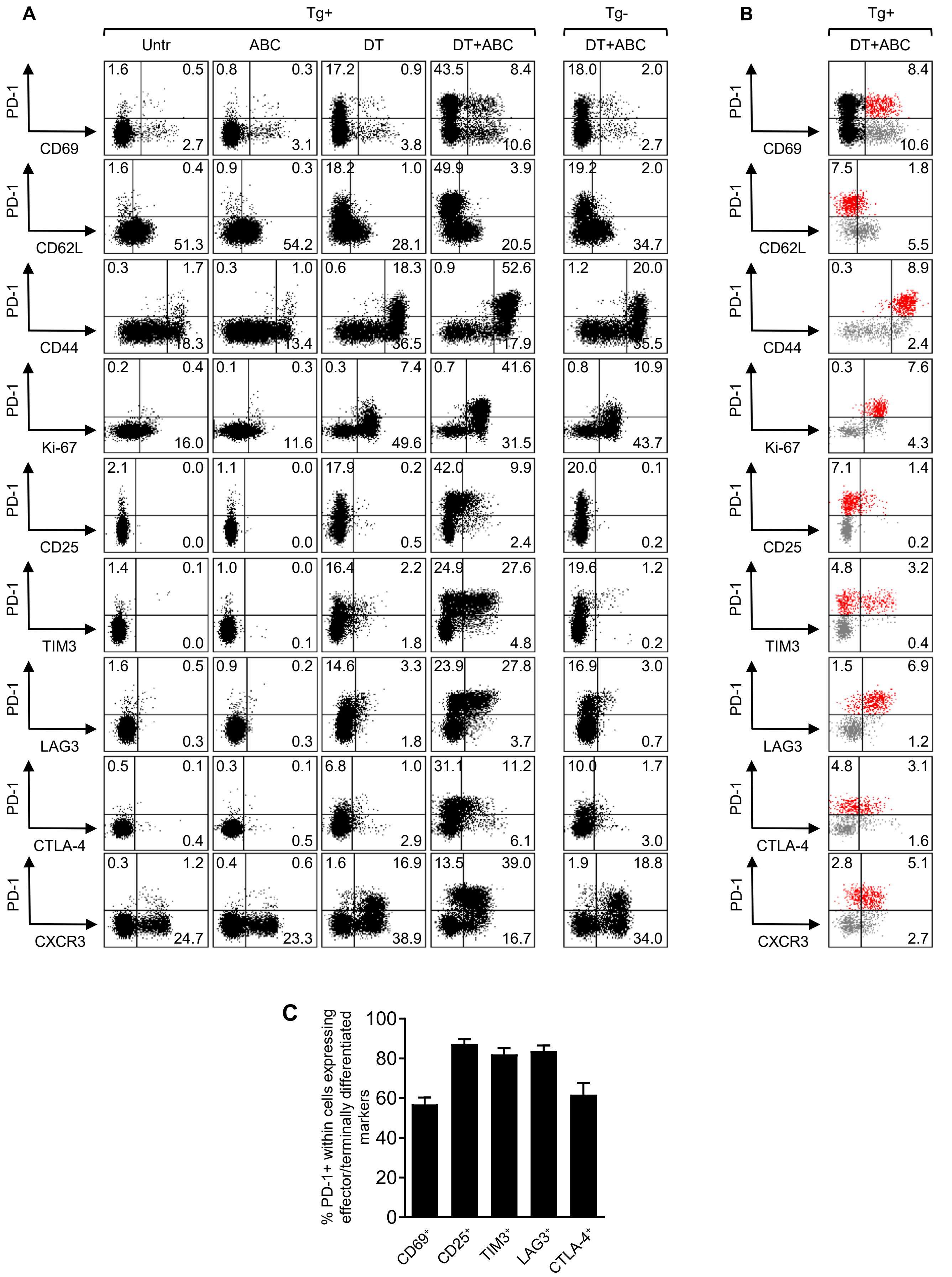

A
Tg+
Untr
ABC
DT
DT+ABC
Tg-
DT+ABC
B
Tg+
DT+ABC
PD-1
CD69
CD62L
CD44
Ki-67
CD25
TIM3
LAG3
CTLA-4
CXCR3
C
% PD-1+ within cells expressing effector/terminally differentiated markers
CD69+
CD25+
TIM3+
LAG3+
CTLA-4+

Supplementary Figure 6: Activation phenotype of PD-1 and CD69-expressing $CD8^+$ T-cells in Spleen at day 8 of treatment. Tg+ or Tg- mice were treated with ABC, DT, or DT+ABC, or left untreated. Spleens were collected at the end of treatment and single cell suspensions were subsequently analyzed by FACS (n=5 mice/group). (A) Representative flow cytometry dot plot with expression levels of activation markers (CD69, CD62L, CD44, CD25, CXCR3), proliferation marker (Ki-67^+), and terminal differentiation markers (TIM3, LAG3, CTLA-4) on $CD8^+$ T-cells . (B) Representative flow cytometry dot plot with the expression levels of activation, proliferation, and terminal differentiation markers on $CD8^+PD\text{-}1^+CD69^+$ (red) and $CD8^+PD\text{-}1^-CD69^+$ (gray) T-cells of DT+ABC-treated Tg+ mice. (C) Summary of levels of expression of PD-1^+ in cells expressing effector/terminally differentiated markers from (B).

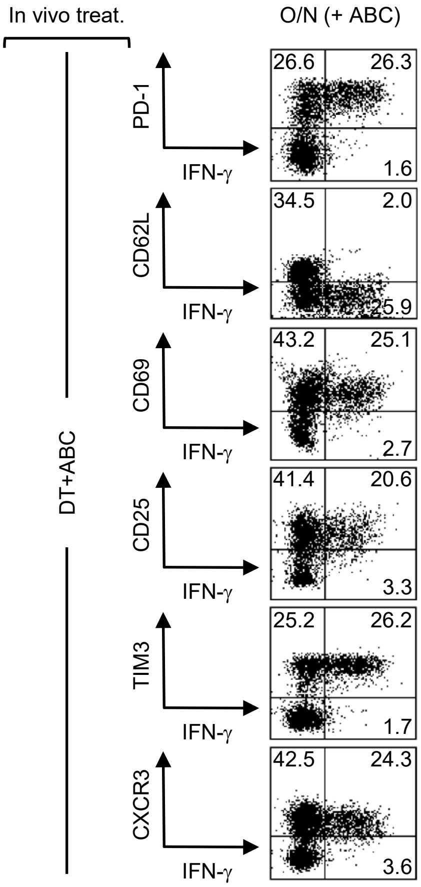

Supplementary Figure 7: Activation status of CD8+ T-cells of DT+ABC animals upon restimulation with ABC in vitro. Spleens from DT+ABC animals treated for 8 days were harvested and prepared for cell restimulation *in vitro* with 10 µg/mL of ABC for 16 hours. Activation and differentiation markers were assessed by cell-surface staining while IFN-γ was evaluated by intracellular staining, followed by flow cytometry analysis. Representative flow cytometry dot plot of an experiment involving 5 mice.

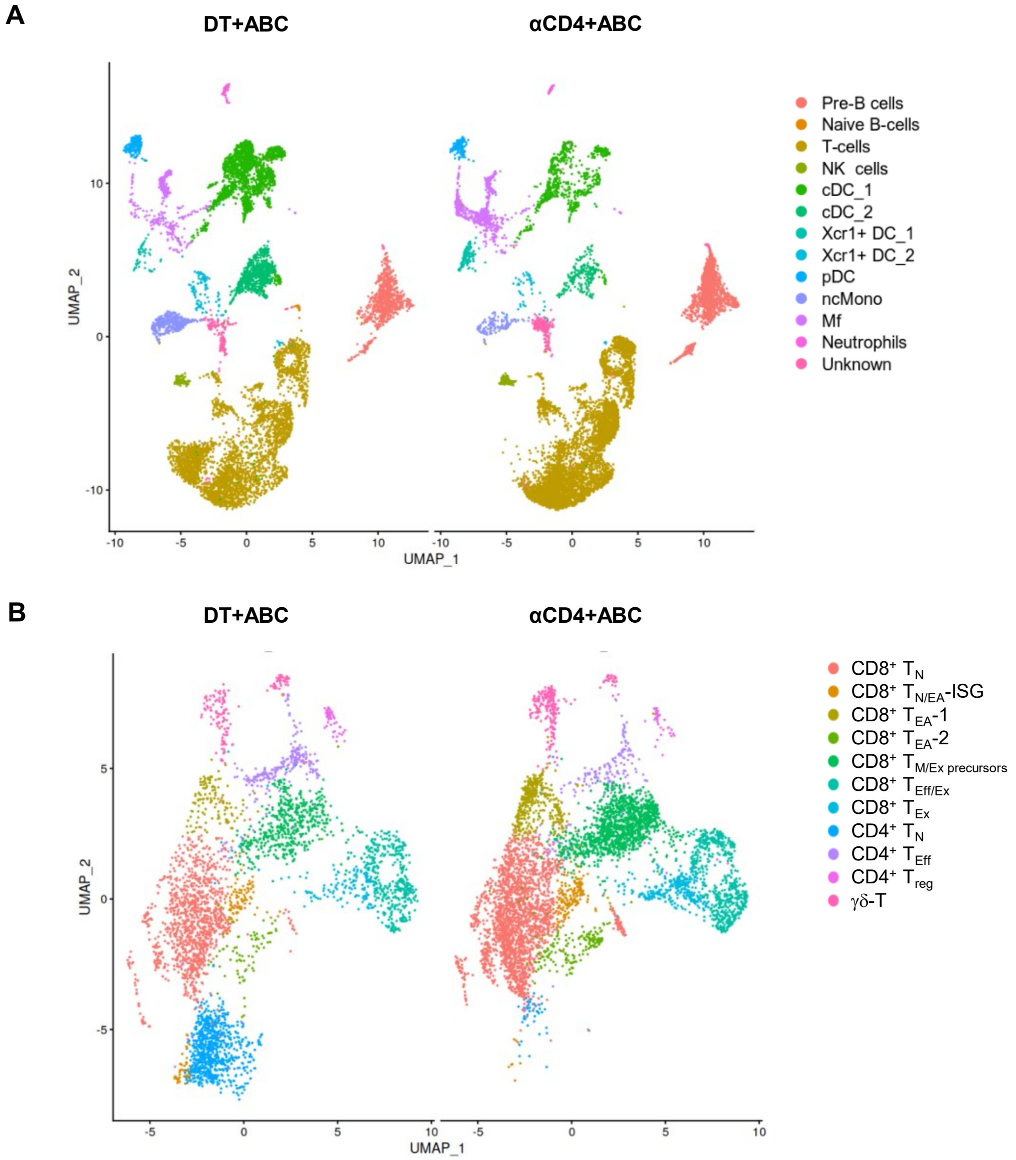

Supplementary Figure 8: Comparable transcriptomic landscape of lymph node cells between αCD4+ABC and DT+ABC treated animals. Lymph nodes (LN) were collected at day 4 of treatment and processed for scRNA-seq analysis as mentioned in Supplementary Methods (n=1/group). Graphs corresponding to DT+ABC LN are borrowed from Figure 5A & C for side-by-side comparability purposes. (A) Cell subtypes including lymphocytes (T, B and NK cells), neutrophils, different subsets of conventional DC (cDC) and Xcr1-expressing DC (Xcr1^{+} DC), macrophages (Mf), plasmacytoid DC (pDC) and non-conventional monocytes (ncMono). (B) Supervised analysis of T-cell cluster in (A) consisting of 11 distinct subsets of CD8^{+}, CD4^{+}, and γδ T-cells with different activation states (naïve (N), early activated (EA), ISG expressing cells (ISG), memory/exhausted precursors (M/Ex precursors), effector (Eff), effector/exhausted (Eff/Ex), and exhausted (Ex)). Reduction of CD4+ cell-related clusters was expected as a result of using αCD4 antibody.

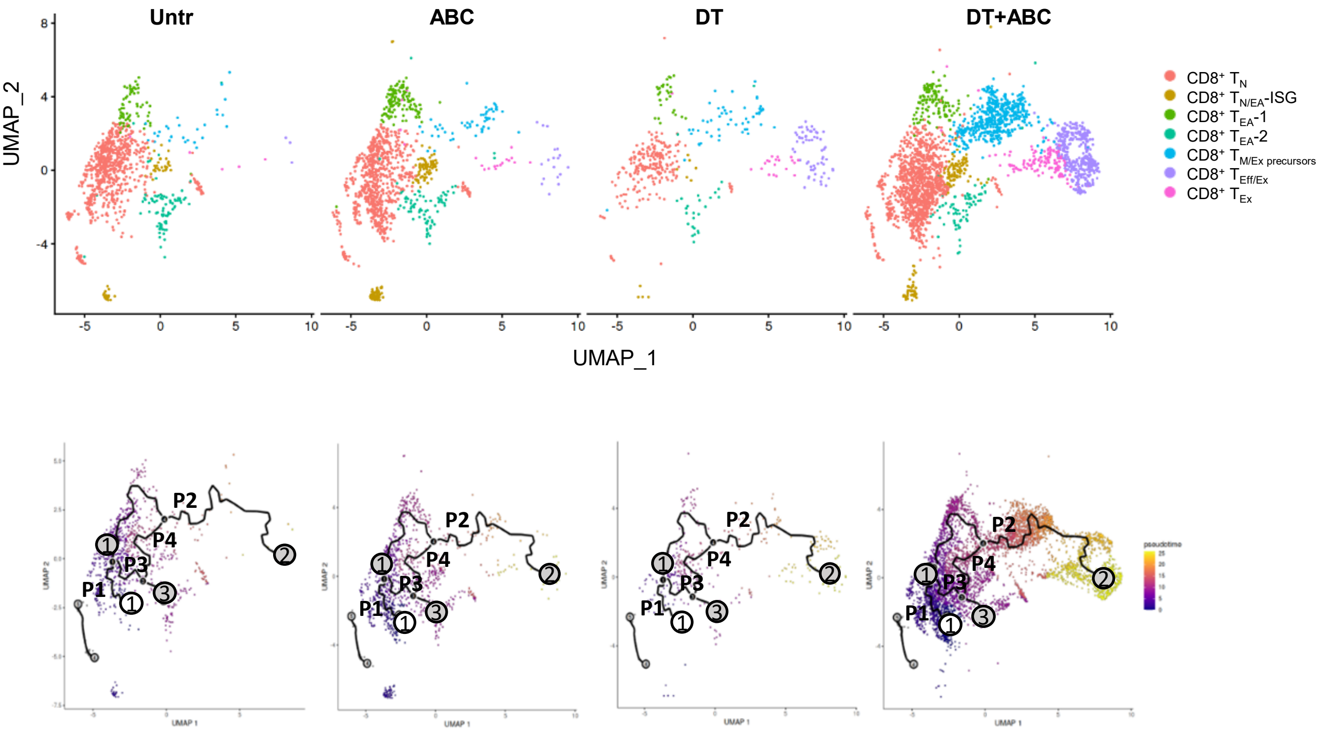

Supplementary Figure 9: Uniform manifold approximation and projection (UMAP) and trajectory analysis of $CD8^+$ T-cell subsets by treatment. Lymph node (LN) cells were collected on day 4 of treatment and processed for scRNA-seq analysis. Top panels represent LN CD8+ T-cell clusters in animals receiving different treatments (n=1/group). Lower panels depict trajectory analysis of $CD8^+$ T-cells above conducted as detailed in the Supplementary Methods. White circle represents the pre-established trajectory origin within the $CD8^+$ T_N. Gray circles correspond to the end point of the paths (P1-4). Black circles correspond to branch points of a given path.
